# *Adenylyl cyclase 3* haploinsufficiency confers susceptibility to diet-induced obesity and insulin resistance in mice

**DOI:** 10.1038/srep34179

**Published:** 2016-09-28

**Authors:** Tao Tong, Ying Shen, Han-Woong Lee, Rina Yu, Taesun Park

**Affiliations:** 1 Department of Food and Nutrition, Brain Korea 21 PLUS Project, Yonsei University, 50 Yonsei-ro, Seodaemun-gu, Seoul 120-749, South Korea; 2 Department of Biochemistry, Yonsei University, 50 Yonsei-ro, Seodaemun-gu, Seoul 120-749, South Korea; 3 Department of Food Science and Nutrition, University of Ulsan, Mugeo-dong, Nam-ku, Ulsan 680-749, South Korea

## Abstract

*Adenylyl cyclase 3 (Adcy3*), a member of the mammalian adenylyl cyclase family responsible for generating the second messenger cAMP, has long been known to play an essential role in olfactory signal transduction. Here, we demonstrated that *Adcy3* heterozygous null mice displayed increased visceral adiposity in the absence of hyperphagia and developed abnormal metabolic features characterized by impaired insulin sensitivity, dyslipidemia, and increased plasma levels of proinflammatory cytokines on both chow and high-fat diet (HFD). Of note, HFD decreased the *Adcy3* expression in white adipose tissue, liver, and muscle. We also report for the first time that *Adcy3* haploinsufficiency resulted in reduced expression of genes involved in thermogenesis, fatty acid oxidation, and insulin signaling, with enhanced expression of genes related to adipogenesis in peripheral tissues of mice. In conclusion, these findings suggest that cAMP signals generated by Adcy3 in peripheral tissues may play a pivotal role in modulating obesity and insulin sensitivity.

Adenylyl cyclases (Adcys), the downstream enzymes for G-protein-coupled receptors (GPCRs), catalyze the conversion of adenosine triphosphate (ATP) into the universal second messenger cyclic AMP (cAMP) which mediates several physiological functions in mammals including embryogenesis, hormone secretion, glycogen breakdown, smooth-muscle relaxation, cardiac contraction, and olfaction^1^,^2^,^3^. Nine isoforms of membrane-bound Adcys are known, each encoded by a distinct gene. Depending on the properties and the relative levels of the isoforms expressed in a tissue or a cell type at a specific time, extracellular signals received through GPCRs can be integrated differently. In the mammalian brain, mRNA for *Adcy1* and *Adcy2* is highly expressed in regions associated with learning and memory, including the hippocampus, cerebral cortex, and cerebellum^1^, while primary cilia of bile cholangiocytes^4^, bone cells^5^, and renal epithelial cells^6^ are known to express *Adcy3*, *Adcy4*, *Adcy6*, and *Adcy8*.

Adcy3 is a 130-kDa glycosylated protein involved in the cascade required for detection of odorants in olfactory neurons^7^. Coupling of odorant receptors to Adcy3 stimulates cAMP transients that function as the major second messengers for olfactory signaling^7^. *Adcy3* knockout mice (*Adcy3*^−/−^) are known to be anosmic (unable to smell) and to have a very high fatality rate after birth. It is believed that the neonatal mortality is due to the inability of pups to smell their mother, compromising their ability to nurse^8^. Besides the well-known function of Adcy3 in regulating olfaction, it has also been suggested to exert ectopic functions based on studies of *Adcy3*-deficient mice^9^,^10^,^11^. Inactivation of the *Adcy3* gene significantly reduced male fertility: spermatozoa from *Adcy3*^−/−^ male mice exhibited decreased motility and showed an increase in spontaneous acrosome reactions^9^,^10^. Moreover, plasma renin and glomerular filtration rates were significantly decreased in *Adcy3*^−/−^ mice, implying that *Adcy3* may play a critical role in regulating fundamental aspects of renal function^11^.

Several lines of evidence suggest the interesting possibility that Adcy3 may play an important role in the regulation of adiposity. For example, genetic association studies of the *Adcy3* in Swedish^12^ and Chinese^13^ populations led to the discovery of *Adcy3* polymorphisms associated with decreased risk of obesity. Furthermore, Wang *et al.* reported that *Adcy3*^−/−^ mice fed chow exhibited higher total fat mass measured by whole body composition analyzer, hyperphagia, and lower physical activity, and speculated that these phenotypic changes conferred by *Adcy3* knockout were associated with disruption of cAMP signaling in primary cilia of the hypothalamus^14^. Pitman *et al.* recently found that the gain-of-function mutation of Adcy3 gene had increased Adcy3 activity and cAMP production and consequently the mutant mice had significantly lower total body weights and fat mass compared to wild type controls after 12 weeks of HFD feeding^15^. It has been established that cAMP signaling, in addition to its involvement in regulating feeding behavior and leptin sensitivity in the hypothalamus^16^,^17^, also has a role in controlling adipose tissue development and function through regulating the expressions of genes related to adipogenesis, lipolysis, and thermogenesis^18^,^19^,^20^. There is also some evidence to suggest Adcy3 may play physiological roles in muscle and liver. For example, Griffin *et al.* found that Adcy3 is expressed in primary muscle cell and is associated with muscle cell migration and adhesion^21^. In addition, hepatic Adcy3 was reported to play a protective role in insulin resistance and obesity in mice with HFD-induced obesity^22^. The present study was aimed at determining whether *Adcy3* functions in peripheral tissues related to metabolic disorders by regulating the development of adiposity and insulin resistance in mice.

## Results

### Adcy3^+/**−**^ mice display increased visceral adiposity

**Figure 1:**
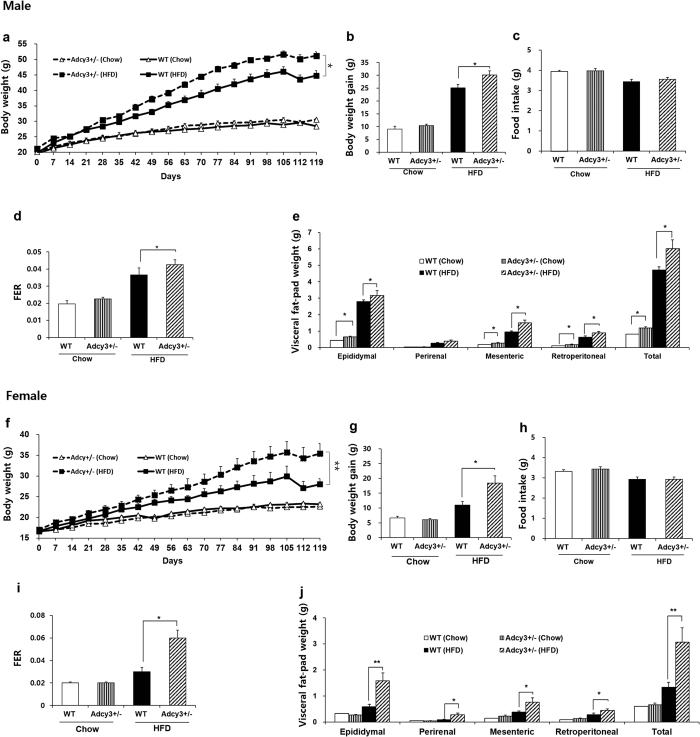
*Adcy3*^+/**−**^ mice are prone to develop visceral adiposity. (**a,f**) Changes in body weight during 17 weeks of feeding. (**b,g**) Cumulative body weight gain. (**c,h**) Daily food intake. (**d,i**) Food efficiency ratio (FER; bodyweight gain over the experimental period [g]/food intake over the experimental period [g]). (**e,j**) Visceral fat-pad weight. Data for both male (upper panels) and female (lower panels) mice are presented. Values are presented as means ± SEM (*n* = 8). Significant differences between groups are indicated by asterisks; *P < 0.05; **P < 0.01; ***P < 0.001.

*Adcy3*^+/**−**^ mice maintained on HFD for 17 weeks exhibited a significant increase in final body weight (14% for male, *p* < 0.05; 26% for female, *p* < 0.01), cumulative body weight gain (20% for male, *p* < 0.05; 63% for female, *p* < 0.05), and total visceral fat-pad weights (27% for male, *p* < 0.05; 133% for female, *p* < 0.01) as compared to WT mice (Fig. 1a,b,e–g,j). There was no significant difference in food intake between *Adcy3*^+/**−**^ and WT mice for male and female (Fig. 1c,h). The food efficiency ratio in *Adcy3*^+/**−**^ mice was significantly higher (16% for male, *p* < 0.05; 100% for female, *p* < 0.05) as compared to WT mice under conditions of HFD feeding (Fig. 1d,i). Male *Adcy3*^+/**−**^ mice fed chow were found to have significantly higher visceral fat-pad weight as compared to WT mice (Fig. 1e).

### Plasma and hepatic biochemical parameters in mice

**Figure 2:**
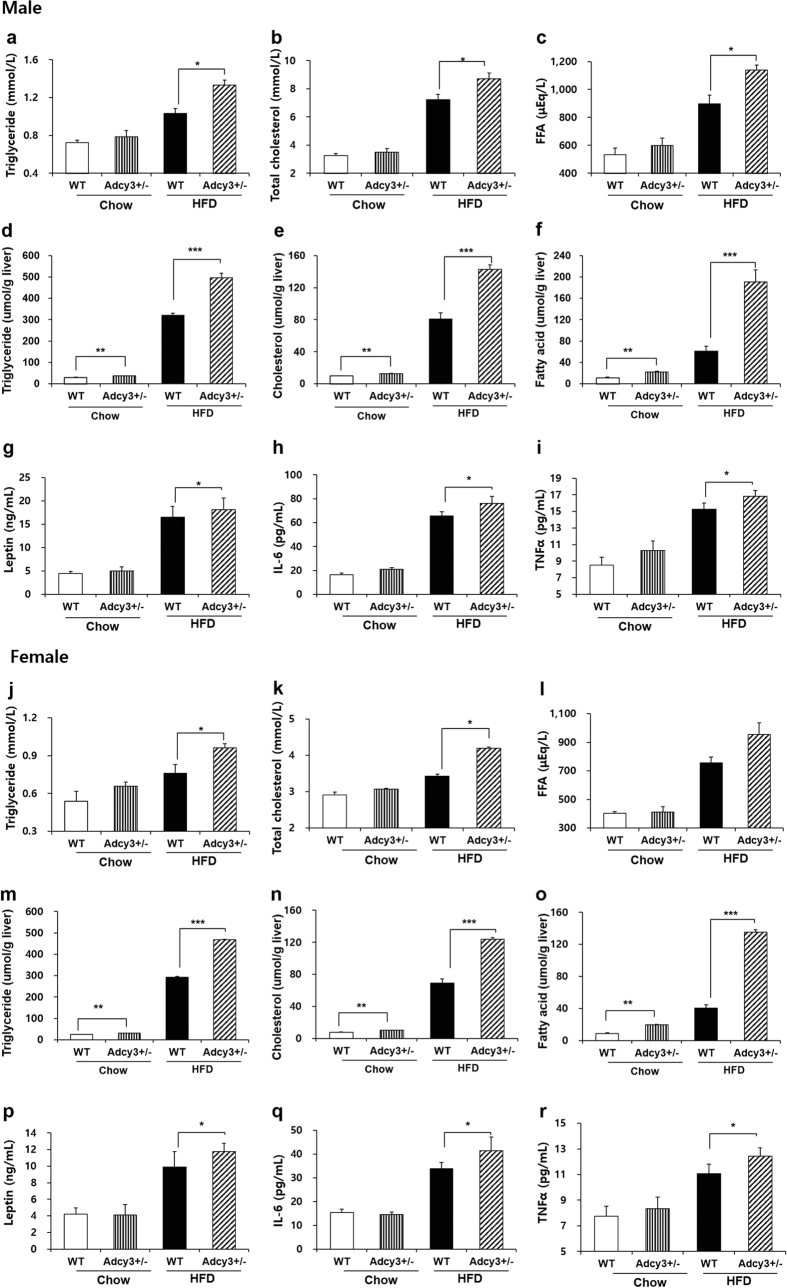
Plasma and hepatic biochemical parameters in mice. Plasma levels of (**a,j**) triglyceride, (**b,k**) total cholesterol, and (**c,l**) FFA. Hepatic contents of (**d,m**) triglyceride, (**e,n**) cholesterol, and (**f,o**) FFA. Plasma levels of (**g,p**) leptin, (**h,q**) IL-6, and (**i,r**) TNFα. Data for both male (upper panels) and female (lower panels) mice are presented. Values are presented as mean ± SEM (*n* = 8). Significant differences between groups are indicated by asterisks; *P < 0.05; **P < 0.01; ***P < 0.001.

*Adcy3*^+/**−**^ mice on HFD exhibited significantly higher levels of plasma TG (29% for male, *p* < 0.05; 26% for female, *p* < 0.05), TC (20% for male, *p* < 0.05; 23% for female, *p* < 0.05), and FFA (27% for male, *p* < 0.05; 26% for female) relative to WT mice (Fig. 2a–c,j–l). Hepatic triglyceride (55% for male, *p* < 0.001; 60% for female, *p* < 0.001), cholesterol (77% for male, *p* < 0.001; 79% for female, *p* < 0.001), and fatty acid (216% for male, *p* < 0.001; 234% for female, *p* < 0.001) levels were significantly higher in *Adcy3*^+/**−**^ mice relative to levels observed in WT mice on HFD (Fig. 2d–f,m–o). *Adcy3*^+/**−**^ mice had significantly higher plasma concentrations of leptin (10% for male, *p* < 0.05; 19% for female, *p* < 0.05), IL-6 (16% for male, *p* < 0.05; 23% for female, *p* < 0.05), and TNFα (10% for male, *p* < 0.05; 12% for female, *p* < 0.05) as compared to WT mice on HFD (Fig. 2g–i,p–r). *Adcy3*^+/**−**^ mice fed chow had significantly higher hepatic triglyceride (23% for male, *p* < 0.01; 25% for female, *p* < 0.01), cholesterol (28% for male, *p* < 0.01; 32% for female, *p* < 0.01), and fatty acid (96% for male, *p* < 0.01; 128% for female, *p* < 0.01) levels than WT mice (Fig. 2d–f,m–o). In the meantime, there was no significant difference in plasma levels of TG, TC, FFA, and proinflammatory cytokines between *Adcy3*^+/**−**^ and WT mice fed chow irrespective of gender (Fig. 2a–c,g–i,j–l,p–r).

### *Adcy3* ^+/**−**^ mice display impaired glucose homeostasis

**Figure 3:**
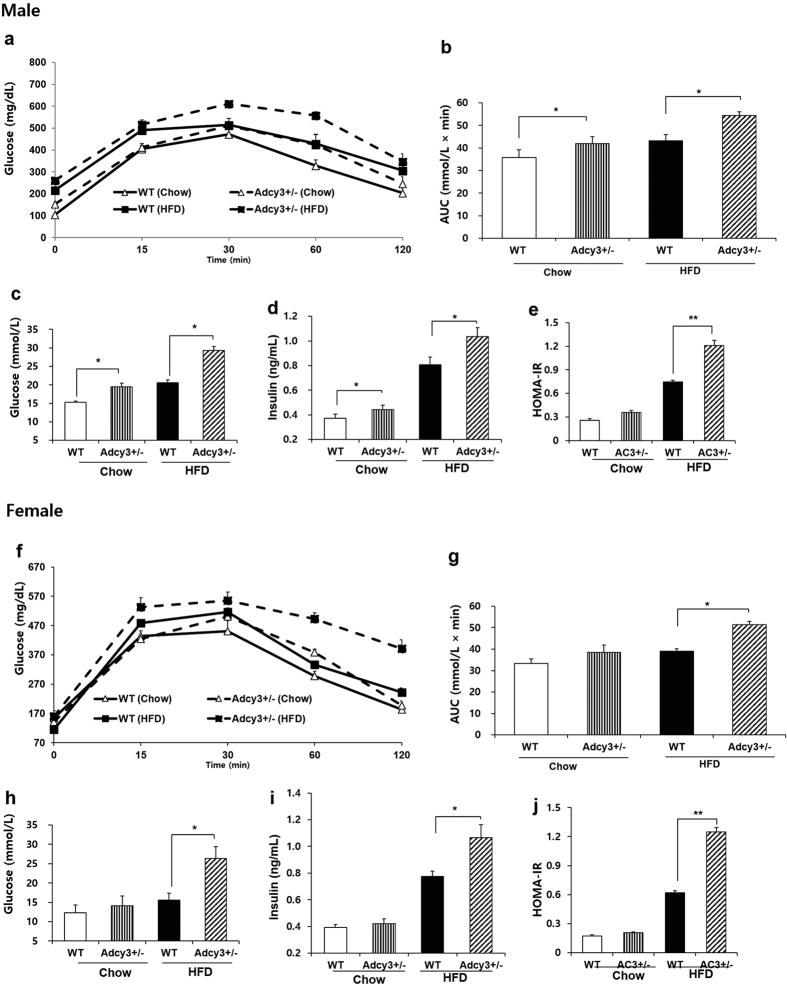
*Adcy3*^+/**−**^ mice have impaired glucose tolerance and are less insulin sensitive. (**a,f**) Oral glucose tolerance test. (**b,g**) Area under the curve. (**c,h**) Fasting plasma glucose levels. (**d,i**) Fasting insulin levels. (**e,j**) Homeostasis model assessment of basal insulin resistance. Data for both male (upper panels) and female (lower panels) mice are presented. Values are presented as means ± SEM (*n* = 8). Significant differences between groups are indicated by asterisks; *P < 0.05; **P < 0.01; ***P < 0.001.

The oral glucose tolerance test (OGTT) was performed 2 weeks prior to the end of the experimental period. Integrated plasma glucose concentration, as calculated by area under the curve (AUC), was increased in *Adcy3*^+/**−**^ mice relative to their WT counterparts on both chow (18% for male, *p* < 0.05; 13% for female) and HFD (26% for male, *p* < 0.05; 32% for female, *p* < 0.05; Fig. 3a,b,f,g). Fasting plasma glucose and insulin levels were measured at the end of the feeding period. *Adcy3*^+/**−**^ mice fed HFD had significantly higher fasting plasma concentrations of glucose (42% for male, *p* < 0.05; 69% for female, *p* < 0.05; Fig. 3c,h) and insulin (28% for male, *p* < 0.05; 38% for female, *p* < 0.05; Fig. 3d,i) compared with WT mice. Along with the plasma glucose and insulin levels, the HOMA-IR values indicated that insulin sensitivity was significantly decreased in *Adcy3*^+/**−**^ mice maintained on HFD (Fig. 3e,j).

**Figure 4:**
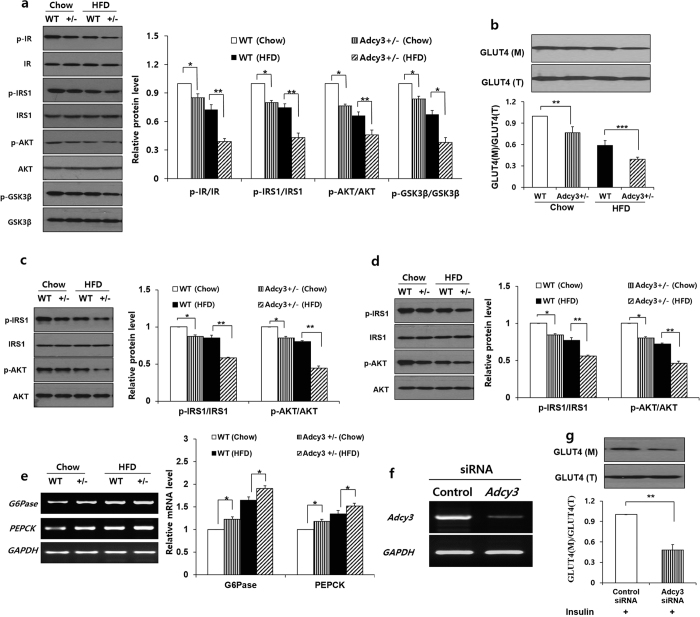
*Adcy3* haploinsufficiency led to impairment of insulin signaling. (**a,b**) Protein levels of p-IR, IR, p-IRS1, IRS1, p-AKT, AKT, p-GSK3β, GSK3β, membrane GLUT4, and total GLUT4 in the epididymal adipose tissue. (**c,d**) Protein levels of p-IRS1, IRS1, p-AKT, and AKT in the (**c**) liver and (**d**) muscle. (**e**) mRNA levels of *G6Pase* and *PEPCK* in the liver of mice. (**f**) The knockdown efficiency by siRNA was monitored by semiquantitative RT-PCR. (**g**) Insulin-stimulated GLUT4 translocation in 3T3-L1 adipocytes treated with control siRNA or siRNA against *Adcy3*. The full-length blots/gels are presented in Supplementary Figs 1–7. Values are presented as means ± SEM (*n* = 8). Significant differences between groups are indicated by asterisks; *P < 0.05; **P < 0.01; ***P < 0.001.

The protein levels of phosphor-IR, phosphor-IRS1, phosphor-AKT, phosphor-GSK3β were significantly decreased in the epididymal adipose tissue of *Adcy3*^+/**−**^ mice relative to WT mice on both chow and HFD (Fig. 4a). The amount of GLUT4 found in the membrane fraction of the epididymal adipose tissue was significantly decreased, whereas the amount of total cellular GLUT4 remained the same in *Adcy3*^+/**−**^ mice when compared with WT mice (Fig. 4b). Similarly, the protein levels of phosphor-IRS1 and phosphor-AKT were significantly decreased in the liver and muscle of *Adcy3*^+/**−**^ mice relative to WT mice on both chow and HFD (Fig. 4c,d). The expression of the key gluconeogenesis enzymes, such as *PEPCK* and *G6pase*, were found to be significantly increased in the liver of *Adcy3*^+/**−**^ mice than in WT mice on both chow and HFD (Fig. 4e).

### Insulin-stimulated GLUT4 translocation is decreased in *Adcy3* deficient 3T3-L1 adipocytes

To test whether Adcy3 affects the insulin-stimulated GLUT4 translocation from cytosol to plasma membrane, we transiently knocked down *Adcy3* using siRNA in 3T3-L1 adipocytes and measured the membrane-bound GLUT4 in the presence of insulin by western blotting. Relative to scrambled siRNA-transfected controls, treatment with siRNA (*Adcy3* SMARTpool) for 48 h achieved significant reductions of *Adcy3* expression level in 3T3-L1 adipocytes (Fig. 4f). The loss of *Adcy3* led to a significant decrease in the amount of plasma membrane GLUT4 after insulin stimulation (Fig. 4g).

### Adcy3 expression in peripheral tissues of mice

**Figure 5:**
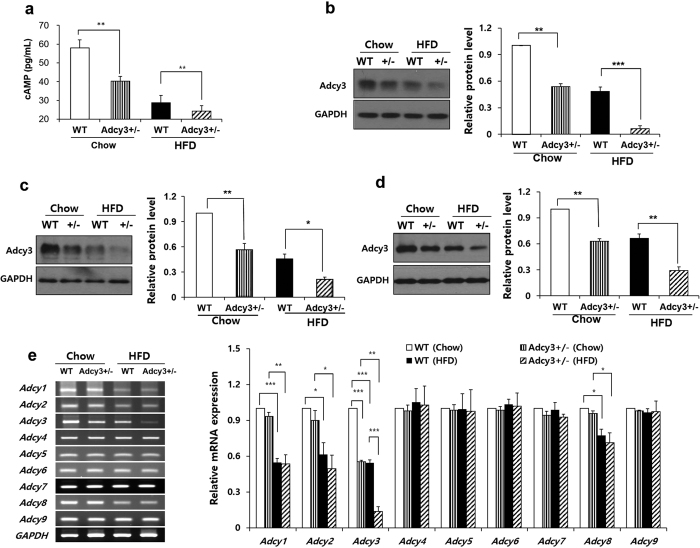
Adcy3 expression in peripheral tissues of mice. (**a**) cAMP concentrations in the epididymal adipose tissue of mice. (**b–d**) Protein level of Adcy3 in the (**b**) epididymal adipose tissue, (**c**) liver, and (**d**) muscle of mice. (**e**) mRNA levels of *Adcy* isoforms in the epididymal adipose tissue of mice. The full-length blots/gels are presented in Supplementary Figs 8–11. Values are presented as means ± SEM (*n* = 8). Significant differences between groups are indicated by asterisks; *P < 0.05; **P < 0.01; ***P < 0.001.

HFD feeding significantly downregulated the mRNA expression of *Adcy1*, *Adcy2*, *Adcy3*, and *Adcy8* in the epididymal adipose tissue of mice but had no effect on the expression of other Adcy isoforms (Fig. 5e). *Adcy3*^+/**−**^ mice showed significantly decreased total Adcy activity in the epididymal adipose tissue as compared to WT mice fed chow (−31%, *p* < 0.01) and HFD (−16%, *p* < 0.01) (Fig. 5a). Both mRNA and protein levels of Adcy3 were significantly reduced in the epididymal adipose tissue of *Adcy3*^+/**−**^ mice than in WT mice irrespective of diet (Fig. 5b,e). The protein level of Adcy3 was also significantly decreased in the liver and muscle of *Adcy3*^+/**−**^ mice relative to WT mice on both chow and HFD. (Fig. 5c,d).

### Altered expression of molecules related to adipogenesis, fatty acid oxidation, and thermogenesis in *Adcy3* ^+/**−**^ mice

**Figure 6:**
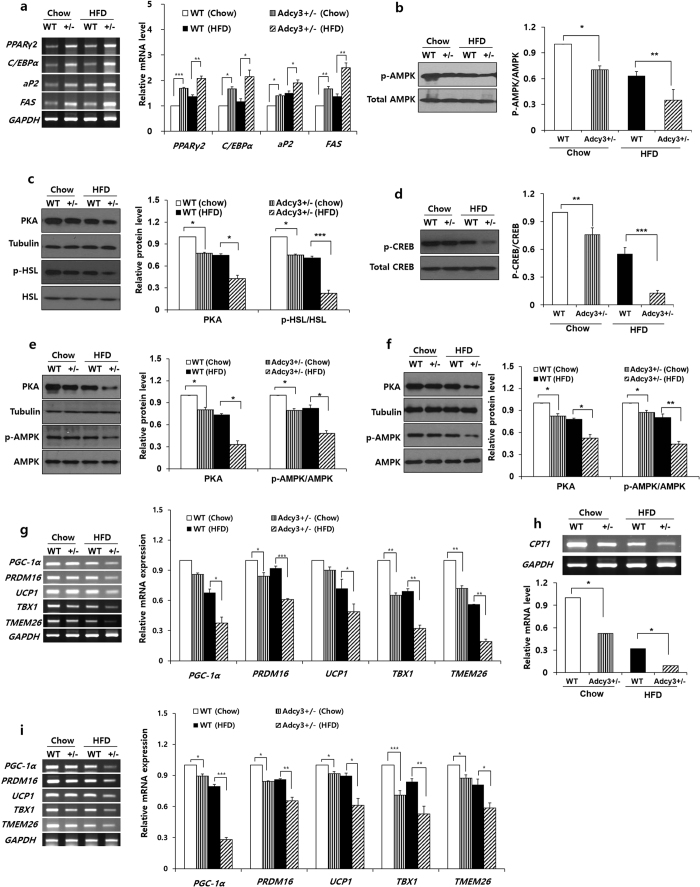
*Adcy3*^+/**−**^ mice display altered expression of genes related to adipogenesis, fatty acid oxidation, and thermogenesis. (**a**) mRNA levels of PPARγ2, C/EBPα, and their target genes in the epididymal adipose tissues of mice. (**b**) Protein levels of p-AMPK and total AMPK in the epididymal adipose tissues of mice. (**c**) Protein levels of PKA, p-HSL, and total HSL in the epididymal adipose tissues of mice. (**d**) Protein levels of p-CREB and total CREB in the epididymal adipose tissues of mice. (**e,f**) Protein levels of PKA and p-AMPK in the (**e**) liver and (**f**) muscle of mice. (**g,h**) mRNA levels of thermogenic genes in the (**g**) epididymal and (**h**) subcutaneous adipose tissues of mice. (**i**) Gene expression of CPT1 in the epididymal adipose tissue of mice. The full-length blots/gels are presented in Supplementary Figs 12–20. Values are presented as means ± SEM (*n* = 8). Significant differences between groups are indicated by asterisks; *P < 0.05; **P < 0.01; ***P < 0.001.

The protein levels of PKA, phosphor-AMPK, phosphor-HSL, and phosphor-CREB were found to be significantly lower in the epididymal adipose tissue of *Adcy3*^+/**−**^ mice than in WT mice on both chow and HFD (Fig. 6b–d). The expression levels of PKA and phosphor-AMPK were also significantly decreased in the liver and muscle of *Adcy3*^+/**−**^ mice compared with WT mice irrespective of diet (Fig. 6e,f). The expression of genes encoding transcription factors, such as peroxisome proliferator-activated receptor *γ2 (PPARγ2*) and CCAAT/enhancer binding protein *α (C/EBPα*), and their targets, adipocyte fatty acid binding protein (*aP2*) and fatty acid synthase (*FAS*), were significantly increased in the epididymal adipose tissue of *Adcy3*^+/−^ mice relative to WT mice on both chow and HFD (Fig. 6a). The mRNA level of carnitine palmitoyl transferase 1 (*CPT1*) were significantly downregulated by *Adcy3* haploinsufficiency in the epididymal adipose tissue of mice on both chow and HFD (Fig. 6h). *Adcy3* haploinsufficiency significantly decreased the expression of thermogenic genes, such as peroxisome proliferator-activated receptor gamma co-activator 1-alpha (*PGC1α*), encoding PR-domain containing 16 (*PRDM16*), uncoupling protein 1 (*UCP1*), T-box transcription factor (*TBX1*), and transmembrane protein 26 (*TMEM26*) in both epididymal and subcutaneous adipose tissues (Fig. 6g,i).

## Discussion

*Adcy3*^+/**−**^ mice (*Adcy3*^*tm1Dgen*^/J) on a 129/OlaHsd background used in the present study were offspring from heterozygous intercross matings of mice originally obtained from the Jackson Laboratory and were found to be healthy and viable. In contrast, homozygous *Adcy3* mice on a 129/OlaHsd background showed a postnatal lethal phenotype^23^, and other homozygous *Adcy3* mice on a 129Sv/J background (*Adcy3*^tm1Drs^) struggled to survive, with an 80% fatality rate within 48 hours, and exhibited abnormal phenotypes, such as anosmia^8^, reduced male fertility^10^, and excretory dysfunction of kidney^11^. In the present study, the increased weight gain and adiposity observed in the *Adcy3*^+/**−**^ mice were not associated with the increased consumption of food, thereby suggesting that *Adcy3* haploinsufficiency does not promote hyperphagia. Consistent with our findings, Pitman *et al.* recently revealed that mice carrying a gain-of-function mutation in *Adcy*3 (*Adcy3*^*Jll/*^+) were protected from diet-induced obesity due to increased oxygen consumption and physical activity without a change in food intake compared to their WT littermates during the 7 days of whole-body metabolic measurements^15^.

In the present study, all membrane-bound *Adcy* isoforms reported, from *Adcy1* through *Adcy9*, were expressed in white adipose tissue (WAT) of mice (Fig. 5). Nevertheless, *Adcy3* appears to be one of the major isoforms contributing to Adcy activity in WAT based on our observation that *Adcy3* haploinsufficiency resulted in a significant decrease (–31%) in total Adcy activity in visceral adipose tissue of mice on chow. Chaudhry *et al.* reported that in brown adipose tissue of rats, the increase in Adcy activity corresponded to a selective upregulation of *Adcy3* expression during the neonatal period when offspring are especially sensitive to environmental conditions and maintenance of body temperature^24^. Moreover, treatment of neonates with the sympathetic neurotoxin 6-hydroxydopamine abolished the perinatal increase in both Adcy activity and *Adcy3* mRNA levels without affecting the expression of other *Adcy* isoforms^24^.

In the present study, the HFD-induced decrease in total Adcy activity observed in visceral adipose tissue of both WT and *Adcy3*^+/**−**^ mice was accompanied by downregulation of *Adcy1*, *Adcy2*, *Adcy3*, and *Adcy8* mRNA expression. This finding is in line with previous reports indicating that expression of *Adcy* isoforms in a specific cell or tissue type can be selectively changed in response to pathophysiologic stimuli^25^,^26^. Choi *et al.* observed that treatment of human colon epithelial cells with butyrate, which induced cell differentiation, downregulated mRNA expression of *Adcy3*, *Adcy4*, *Adcy6,* and *Adcy7* by 70–90%, while mRNA levels of the other three isoforms, including *Adcy1*, *Adcy5*, and *Adcy9*, were unchanged^25^. Furthermore, Suzuki *et al.* reported that among four isoforms of *Adcys (Adcy2*, *Adcy6*, *Adcy7*, and *Adcy*9) detected in the gastrocnemius muscle, *Adcy*2 and *Adcy*9 mRNA were selectively downregulated after denervation carried out by excision of the left sciatic nerve at the midthigh region^26^.

**Figure 7:**
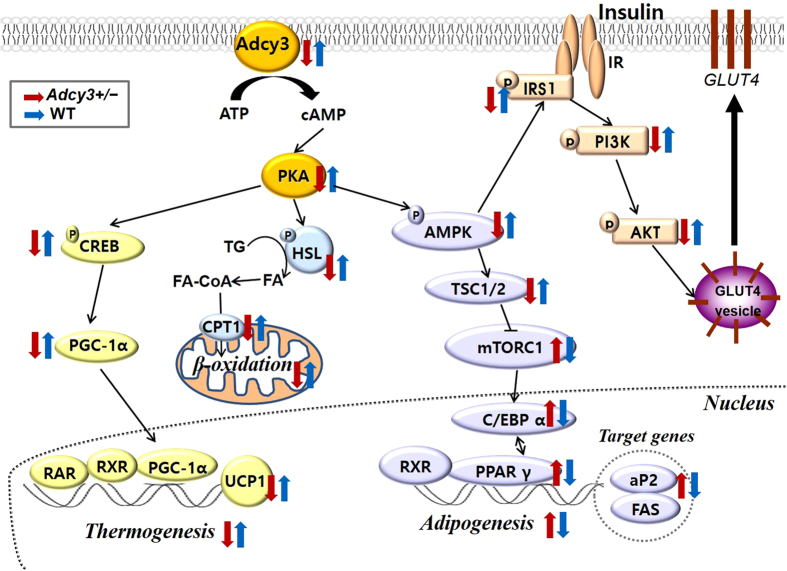
Schematic presentation of the Adcy3-mediated signaling pathways related to adipogenesis, thermogenesis, fatty acid oxidation, and insulin resistance.

The cAMP signaling pathways are pivotal in regulation of adipose tissue development and function^27^. The accumulation of cAMP activates PKA in adipocytes of all colors and origins, thereby phosphorylating several proteins, such as CREB^20^, AMPK^28^, and HSL^29^ (Fig. 7). Phosphor-CREB then activates the expression of PGC-1α, which induces the transcription of downstream thermogenic genes, including UCP1^20^. In parallel, phosphorylated AMPK inhibits preadipocyte differentiation by downregulating PPARγ and C/EBPα, which are the central regulators of adipogenesis and lipid storage in adipocytes^30^. Moreover, PKA-mediated phosphorylation induces translocation of the HSL to the surface of lipid droplets and enhances its catalytic activity^29^. The released free fatty acids are oxidized to fatty acyl-CoA which combines with CPT1 to enter mitochondria for its oxidation. In the present study, *Adcy3* haploinsufficiency significantly decreased the protein levels of PKA, phosphor-CREB, phosphor-HSL, and phosphor-AMPK in WAT of mice fed HFD (Fig. 7). These results indicated that in WAT, Adcy3 might be associated with the regulation of cAMP-PKA-mediated signaling pathways that affect thermogenesis, fatty acid oxidation, and adipogenesis.

Here, *Adcy3* haploinsufficiency reduced insulin sensitivity in mice, as demonstrated by as demonstrated by impairment of insulin signaling in WAT, liver, and muscle, along with increased AUC values and elevated fasting plasma glucose and insulin levels. This insulin resistance observed in *Adcy3*^+/**−**^ mice appears to be associated with decreased AMPK activity (Fig. 7). Reduced AMPK activity is known to decrease the phosphorylation of IRS-1 at Ser789, thus negatively regulating insulin signaling in adipocytes^31^. Moreover, *Adcy3*^+/**−**^ mice showed higher plasma levels of proinflammatory cytokines, such as IL-6 and TNFα, relative to their WT littermates on both chow and HFD. It is well established that within adipose tissue, IL-6 and TNFα cause adipocyte insulin resistance through inactivation of both the insulin receptor and IRS-1, both of which result in diminished activation of phosphoinositol-3-kinase, the essential second messenger signal that governs most of metabolic effects associated with insulin^32^,^33^. Therefore, the increased plasma levels of proinflammatory cytokines could also be a contributing factor for the aggravated insulin resistance in *Adcy3*^+/**−**^ mice.

Possible application of Adcy3 as a target for insulin resistance and anti-obesity treatment has been implicated in a recent study by Liang *et al.*^22^ revealing that hepatic Adcy3 is upregulated in mice with HFD-induced obesity by liraglutide, a glucagon-like peptide-1 analogue recently approved by the US Food and Drug Administration as an obesity treatment option. In conclusion, the *Adcy3* heterozygous null mice displayed increased visceral adiposity in the absence of hyperphagia and developed abnormal metabolic features on both chow and HFD. We report for the first time that HFD decreased the *Adcy3* expression in white adipose tissue, liver, and muscle and that *Adcy3* haploinsufficiency resulted in reduced expression of genes involved in thermogenesis, fatty acid oxidation, and insulin signaling, with enhanced expression of genes related to adipogenesis in peripheral tissues of mice. Although the role of *Adcy3* in humans has yet to be elucidated, our findings raise the possibility that Adcy3 activators may be useful agents for the prevention or treatment of obesity and associated metabolic complications.

## Methods

### Animals and diets

*Adcy3*^+/**−**^ mice (B6.129P2-*Adcy3*^*tm1Dgen*^/J; stock 005773) were generated by Deltagen Inc. (San Mateo, CA, USA) and acquired from the Jackson Laboratories (Bar Harbor, ME, Maine, USA) through the “NIH initiative supporting placement of Deltagen, Inc., mice into public repositories.” Heterozygous intercross was performed to produce *Adcy3*^+/**−**^ mice and wild-type (WT) littermate control mice. Genotyping was performed using PCR, with genomic DNA extracted from tail tips. Three primers were used: WT (5′-CGT CTT CCT CTA CCT GTG TGC TAT C-3′), mutant (5′-GGG CCA GCT CAT TCC TCC CAC TCA T-3′), and common (5′-TCC TAA CGG ACT TAC ACT GAG GTA G-3′).

Four-week-old male and female *Adcy3*^+/**−**^ or WT littermate mice were housed in a pathogen-free facility at 21 ± 2.0 °C, with 50 ± 5% relative humidity and a 12-h light/dark cycle. The mice were provided access to rodent chow and tap water *ad libitum* for 1 week. Thereafter, the mice (*n* = 8) were placed on chow or HFD for 17 weeks. The HFD consisted of 200 g fat/kg body weight (170 g of lard and 30 g of corn oil) and 1% (w/w) cholesterol (supplementary Table 1).

Food intake and body weight were measured daily and weekly, respectively. At the end of the experimental period, the mice were anesthetized with diethyl ether after they were fasted for 16 h. Blood samples were drawn from the inferior vena cava into an ethylene-diamine-tetra-acetic acid (EDTA)-coated tube, and the plasma samples were obtained by centrifuging the blood at 4,000 *g* for 15 min at 4 °C. The epididymal, retroperitoneal, mesenteric, perirenal, and subcutaneous fat-pads were dissected, removed, weighed, and immediately snap-frozen in liquid nitrogen and stored at –80 °C until further use. All animal experiments were performed in accordance with the Korea Food and Drug Administration guidelines. All experimental protocols were reviewed and approved by the Institutional Animal Care and Use Committee of the Yonsei Laboratory Animal Research Center (Permit no. 2011-0062).

### Cell culture and transfection

3T3-L1 fibroblasts obtained from American Type Culture Collection (Manassas, VA, USA) were grown in Dulbecco’s modified Eagle’s medium supplemented with 10% fetal bovine serum, penicillin (50 units/mL), and streptomycin (50 mg/mL). The cells were grown at 37 °C in a humidified 5% CO_2_ atmosphere. We treated confluent cultures with 0.5 mM 3-isobutyl-1-methylxantine, 0.25 mM dexamethasone, and 10 mg/mL insulin to promote the differentiation of 3T3-L1 cells into adipocytes. After 2 days, the 3-isobutyl-1-methylxanthine and dexamethasone were removed, and insulin was continued for another 2 days. The growth medium was replenished at 2-day intervals until adipocyte differentiation. For *Adcy3* knockdown, the differentiated adipocytes were treated with 50 nM of either control siRNA (nontargeting SMARTpool, catalog no. D-001810-10-05, Dharmacon, Lafayette, CO, USA) or siRNA against *Adcy3* (On-TARGET plus SMART pool siRNA, catalog no. L-0588903-00-10, Dharmacon) on the ninth day of differentiation. Two days post-transfection, serum–starved cells were treated with 17 nM insulin for 15 min and then lysed for RNA or protein. The knockdown efficiency by siRNA was monitored by semiquantitative RT-PCR.

### Biochemical analysis

Plasma content of total cholesterol (TC), triglyceride (TG), free fatty acid (FFA), and glucose were enzymatically determined using individual commercial kits (Bio-Clinical System, Gyeonggi-do, South Korea). Plasma concentration of insulin was measured using a commercially available mouse enzyme-linked immunosorbent assay (ELISA) kit (Millipore, Billerica, MA, USA). The homeostasis model assessment of basal insulin resistance (HOMA-IR) was calculated as [fasting plasma glucose × fasting plasma insulin/22.5] to assess insulin resistance. Hepatic lipids were extracted as described using the method developed by Folch *et al.*^34^. Triglyceride, cholesterol, and FFA levels in the hepatic lipid extracts were measured using the same enzymatic kits that were used for the plasma analyses.

### Oral glucose tolerance test

The OGTT was performed 2 weeks before the end of the experimental period on 6 h-fasted mice by oral glucose administration (gavage with 2 g glucose/kg body weight). Blood glucose was measured from tail blood at times 0, 15, 30, 60, 90, and 120 min after glucose administration.

### Western blotting analysis

To obtain total protein, the liver, muscle, and epididymal and subcutaneous adipose tissue samples obtained from each mouse were homogenized at 4 °C in an extraction buffer containing 100 mmol/L Tris-HCl (pH 7.4), 5 mmol/L EDTA, 50 mmol/L NaCl, 50 mmol/L sodium pyrophosphate, 50 mmol/L NaF, 100 mmol/L orthovanadate, 1% Triton X-100, 1 mmol/L phenylmethanesulfonyl fluoride, 2 μg/mL aprotinin, 1 μg/mL pepstatin A, and 1 μg/mL leupeptin, and centrifuged at 13,000 *g* for 20 min at 4 °C. To obtain membrane protein, the epididymal and subcutaneous adipose tissue samples were homogenized in a buffer containing 20 mmol/L HEPES (pH 7.4), 4 mmol/L EDTA, 250 mmol/L sucrose, two tablets of protein inhibitor, 1 mmol/L sodium orthovanadate, and 1% Triton X-100. The homogenates were centrifuged at 2,000 g for 1.5 h at 4 °C, and the supernatant fractions were centrifuged at 150,000 g for 1.5 h at 4 °C. Protein concentrations were measured by Bradford assay (Bio-Rad, Hercules, CA, USA).

Total protein or membrane protein (40 μg) was separated by 8% sodium dodecyl sulfate (SDS)-polyacrylamide gel electrophoresis (PAGE) and then electrophoretically transferred to nitrocellulose membranes (Amersham, Buckinghamshire, UK). The nitrocellulose membranes were incubated overnight with primary antibodies (diluted 1:1,000) at 4 °C. Antibodies to the following proteins were commercially obtained from the indicated sources: β-actin and Adcy3 from Santa Cruz Biotechnology (Santa Cruz, CA, USA) and AMP-activated protein kinase (AMPK), phosphor-AMPK (Thr172), cyclic AMP-responsive element-binding protein (CREB), phosphor-CREB (Ser133), PKA, hormone-sensitive lipase (HSL), phosphor-HSL (Ser563), insulin receptor β subunit (IRβ), phosphor-IRβ (Tyr1162/1163), insulin receptor substrate 1 (IRS1), phosphor-IRS1 (Ser789), protein kinase B (AKT), phosphor-AKT (Ser473), glycogen synthase kinase-3β (GSK3β), phosphor-GSK3β (Ser9), and glucose transporter type 4 (GLUT4) from Cell Signaling Technology (Danvers, MA, USA). Secondary antibodies were applied for 1 hr at room temperature in Tris-buffered saline containing 0.05% Tween-20. Signal was detected by using a chemiluminescent detection system (Amersham). The protein bands were quantified using Quantity One Analysis Software (Bio-Rad).

### Adcy enzymatic assay

Fifty microliters of Adcy mixture [100 mM Tris-acetate (pH 7.4), 20 mM KCl, 10 mM MgCl_2_, 20 mM phosphoenolpyruvate, 100 μg/mg pyruvate kinase, 2 mM ATP, 20 μM guanosine triphosphate, 2 mM dithiothreitol, 0.4 mM bovine serum albumin, and 0.1 mM 3-isobutyl-1-methylxanthine] and 125 μg of membrane fractions from epididymal adipose tissue were added into microcentrifugation tubes in duplicate and maintained at 4 °C. The reaction tubes were incubated in a water bath maintained at 37 °C for 30 min, and the reaction was terminated by heating at 95 °C for 5 min. The supernatant solution was stored after centrifugation at 10,000 g for 5 min at 4 °C. The cAMP assay was performed using the cAMP ELISA kit from Enzo Life Sciences International, Inc. (Plymouth Metting, PA, USA).

### RNA extraction and semi-quantitative reverse transcriptase-polymerase chain reaction (RT-PCR)

Total RNA was isolated from the liver, epididymal, and subcutaneous adipose tissue of each mouse by using TRIzol (Invitrogen, Carlsbad, CA, USA) and was reverse-transcribed by using the Superscript II Kit (Invitrogen) according to the manufacturer instructions. The PCR was programmed as follows: 10 min at 94 °C, 30–35 cycles of 94 °C for 30 s, 55 °C for 30 s, 72 °C for 1 min, and 10-min incubation at 72 °C. Then, 5 μL of each PCR product was mixed with 1 μL of 6-fold concentrated loading buffer and electrophoresed on a 2% agarose gel containing ethidium bromide. The band intensities were quantified using the Quantity One Analysis Software (Bio-Rad). The mRNA levels were normalized to that of the glyceraldehyde-3-phosphate dehydrogenase transcript. Sequences of all primers are listed in supplementary Table 2.

### Statistics

Results on body weight gain and plasma biochemistries are presented as the mean ± standard error of mean (SEM) of eight mice in each group. The RT-PCR and western blot data are shown as the means ± SEM of three independent experiments (*n* = 2 or 3 per experiment) for each group, cumulatively including eight mice. An unpaired Student *t*-test analysis was used for all data comparisons between the *Adcy3*^+/**−**^ and WT control mice fed chow or HFD. All statistical analyses were performed with SPSS 21.0 software (IBM, Corp., Armonk, NY, USA), and significance was set at *P < 0.05, **P < 0.01 and ***P < 0.001.

## Additional Information

**How to cite this article**: Tong, T. *et al.* *Adenylyl cyclase 3* haploinsufficiency confers susceptibility to diet-induced obesity and insulin resistance in mice. *Sci. Rep.* **6**, 34179; doi: 10.1038/srep34179 (2016).

## Supplementary Material

Supplementary Information
