# Supplementary material for: Adenylyl cyclase 3 haploinsufficiency confers susceptibility to diet-induced obesity and insulin resistance in mice
Source: Sci Rep. 2016 Sep 28;6:34179. doi: 10.1038/srep34179 (PMC5039768; doi:10.1038/srep34179)
Supplement: Supplementary Information [file srep34179-s1.pdf]

Supplementary Information

Adenylyl cyclase 3 haploinsufficiency confers susceptibility to diet-induced obesity and insulin resistance in mice

Tao Tong[1], Ying Shen[1], Han-Woong Lee[2], Rina Yu[3], and Taesun Park[1*]

[1]*Department of Food and Nutrition, Brain Korea 21 PLUS Project, Yonsei University, 50 Yonsei-ro, Seodaemun-gu, Seoul 120-749, South Korea.*

[2]*Department of Biochemistry, Yonsei University, 50 Yonsei-ro, Seodaemun-gu, Seoul 120-749, South Korea.*

[3]*Department of Food Science and Nutrition, University of Ulsan, Mugeo-dong, Nam-ku, Ulsan 680-749, South Korea.*

[*]Correspondence: Taesun Park, Department of Food and Nutrition, Yonsei University, 50 Yonsei-ro, Seodaemun-gu, Seoul 120-749, South Korea. Phone: +82-2-2123-3123; Fax: +82-2-365-3118; E-mail: tspark@yonsei.ac.kr

Supplementary Table 1. Composition of high-fat diet and standard chow diet (Purina Laboratory Rodent Diet 5001, LabDiet).

	**HFD (4.6 Kcal/g)**	**Chow (3.35 Kcal/g)**
**Nutrient Information**	% kcal from	% kcal from
Protein	18	28.5
Carbohydrate	42	58
Fat	40	13.5
**Formula**	**g/Kg**	**Representative Ingredients**
Casein	200	Dehulled soybean meal
DL-methionine	3	DL-methionine
Corn starch	111	Ground corn
Sucrose	370	Dried beet pulp
Cellulose	50	Wheat middlings
Corn oil	30	Cane molasses
Lard	170	Fish meal
Cholesterol	10	Porcine fat preserved with BHA
Mineral mixture[1)]	42	Ground oats
Vitamin mixture[2)]	12	Dehydrated alfalfa meal
Choline bitartrate	2	Choline chloride
tert- Butyhydeoquinone[3)]	0.04	Brewers dried yeast

[1)] AIN-76A mineral mix.
[2)] AIN-76A vitamin mix.
[3)] Antioxidant agent: 0.01 g/ 50 g lipids.

Supplementary Table 2. Primer sequences

Gene description	Sequences (5′→3)	Size(bp)
Adenylyl cyclase 1 (*Adcy1*)	F: AAGTCTGGCCCAAGCAGATT<br>R: CGGGGTGTGTGTGTACCAAT	150
Adenylyl cyclase 2 (*Adcy2*)	F: TGGATGGAGCCAAGATGAGA<br>R: GCACATCCGTGGTGCTAATC	138
Adenylyl cyclase 3 (*Adcy3*)	F: GTGCTATCATCGTGGGCATC<br>R: TCCTTCAGCATCTCGTCAGC	178
Adenylyl cyclase 4 (*Adcy4*)	F: TGGTGGCATCTTGTTCCCTA<br>R: CCAGGCGGCAGTAATACTCA	198
Adenylyl cyclase 5 (*Adcy5*)	F: TGCCTGTGTGAAGCTTTTCC<br>R: GAACACCAGGGTGATGGTGA	109
Adenylyl cyclase 6 (*Adcy6*)	F: ACGTTGCCATGGAGATGAAA<br>R: TCAATGTCCGCAAACAGGAT	112
Adenylyl cyclase 7 (*Adcy7*)	F: ATGACAGGTCAATGCTCCGA<br>R: AAAAGGAGCCAGGCACTGAT	128
Adenylyl cyclase 8 (*Adcy8*)	F: GAAGATTTCCTGGGGACCAA<br>R: TGGGTAAGATATTGCGCAGC	196
Adenylyl cyclase 9 (*Adcy9*)	F: CAGGAGCTGGAGCGATCATA<br>R: TCCAGAAGGGAGCTGAAGGT	101
Peroxisome proliferator-activated receptor γ2 (*PPARγ2*)	F: TTCGGAATCAGCTCTGTGGA<br>R: CCATTGGGTCAGCTCTTGTG	170
CCAAT/enhancer binding-protein α (*C/EBP α*)	F: TCAGCTTACAACAGGCCAGG<br>R: ACACAAGGCTAATGGTCCCC	174
Adipocyte fatty acid binding protein (*aP2*)	F: CATGCGACAAAGGCAGAAAT<br>R: GTTACAAGGCAAGGAAGGGC	163
Fatty acid synthase (*FAS*)	F: CAGCCAGGAGAATCGCAGTA<br>R: CTGCGATGAAGAGCATGGTT	153
Carnitine palmitoyl transferase 1 (*CPT1*)	F: AGGTATGGCCACTTTGGGAC<br>R: GCTGGTCTTGCTGTGCATCT	159
Peroxisome proliferator-activated receptor gamma coactivator 1-alpha (*PGC1α*)	F: TAAATCTGCGGGATGATGGA<br>R: GTTTCGTTCGACCTGCGTAA	109
Uncoupling protein 1 (*UCP1*)	F: GGTTTTGCACCACACTCCTG<br>R: ACATGGACATCGCACAGCTT	108
PR domain containing 16 (*PRDM16*)	F: GGACCTTTTTGACAGCAGCA<br>R: GGGGGCAAAGCATTTAACTC	119
T-box transcription factor (*TBX1*)	F: CGAGATGATCGTCACCAAGG<br>R: TACCGGTAGCGCTTGTCATC	132
Transmembrane protein 26 (*TMEM26*)	F: ACAGTACACGTGAAGCCCCA<br>R: GGTCACGGGGTTTTCTCACT	141
Phosphoenolpyruvate carboxykinase (*PEPCK*)	F: CACCTCCTGGAAGAACAAGG<br>R: CTA CGGCCACCAAAGATGAT	161
Glucose-6-phosphatase (*G6Pase*)	F: GTCGACTCGCTATCTCCAAG<br>R: GCAATGCCTGACAAGACTCC	528
Glyceraldehyde-3-phosphate dehydrogenase (*GAPDH*)	F: GTCGTGGAGTCTACTGGTGT<br>R: TGCTGACAATCTTGAGTGAG	183

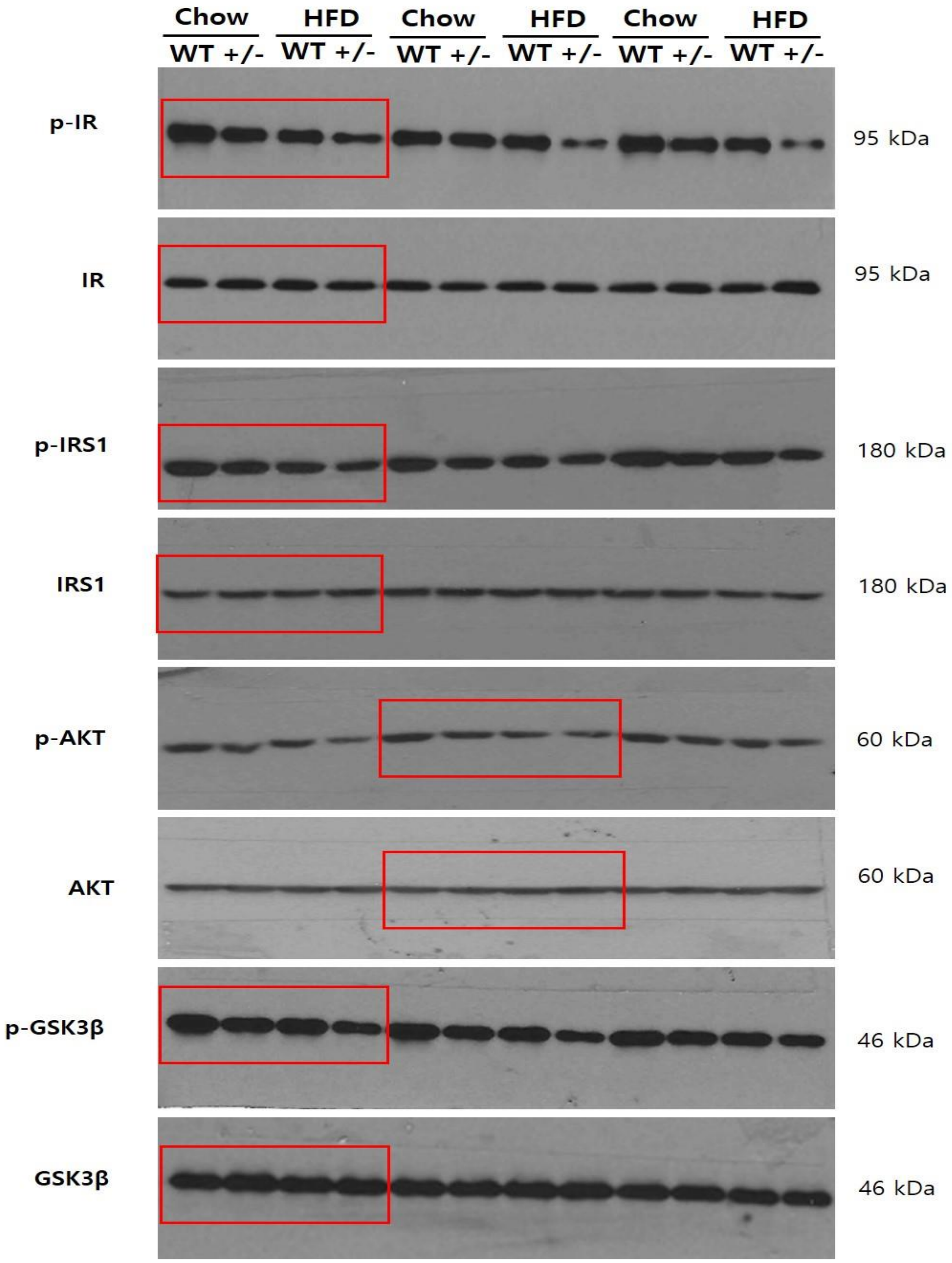

Supplementary Figure S1. Full length western blot membranes relative to Fig 4a. Red boxes indicate the cropping lines used to generate the figures.

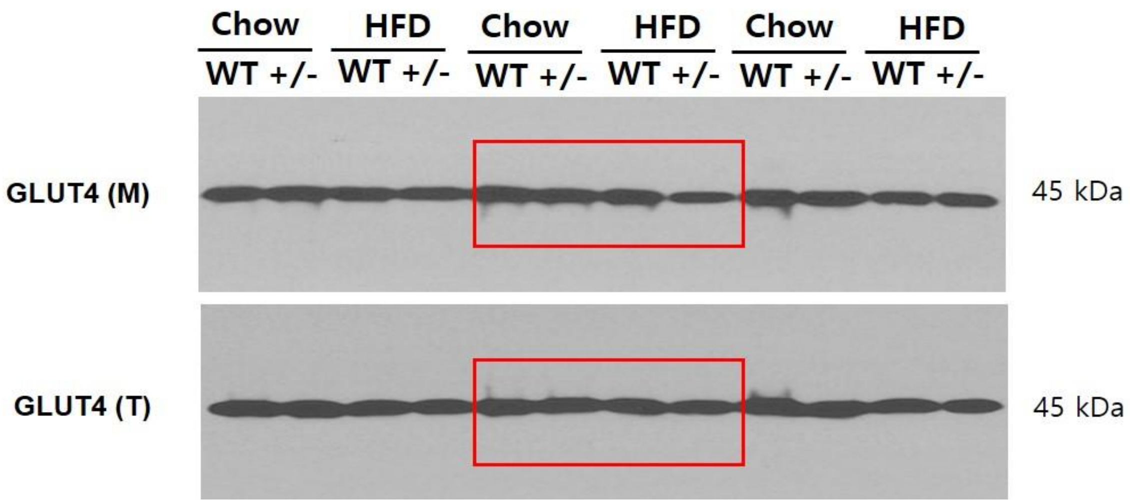

Supplementary Figure S2. Full length western blot membranes relative to Fig 4b. Red boxes indicate the cropping lines used to generate the figures.

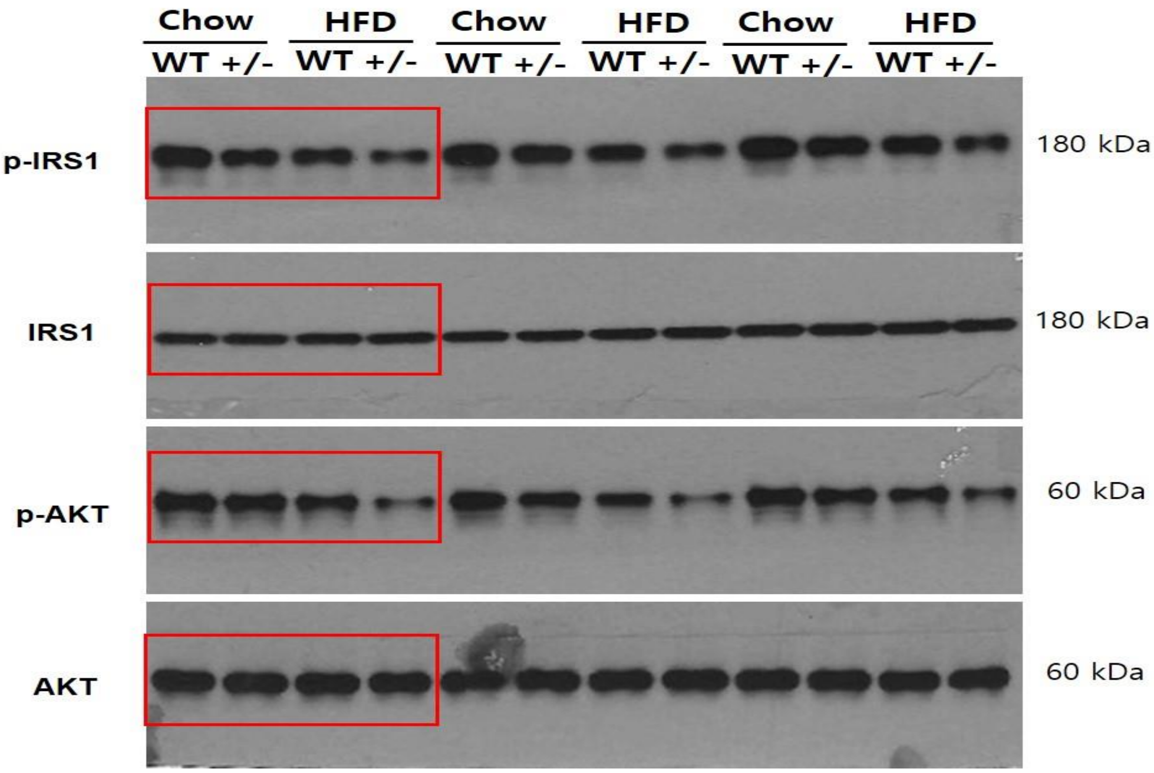

Supplementary Figure S3. Full length western blot membranes relative to Fig 4c. Red boxes indicate the cropping lines used to generate the figures.

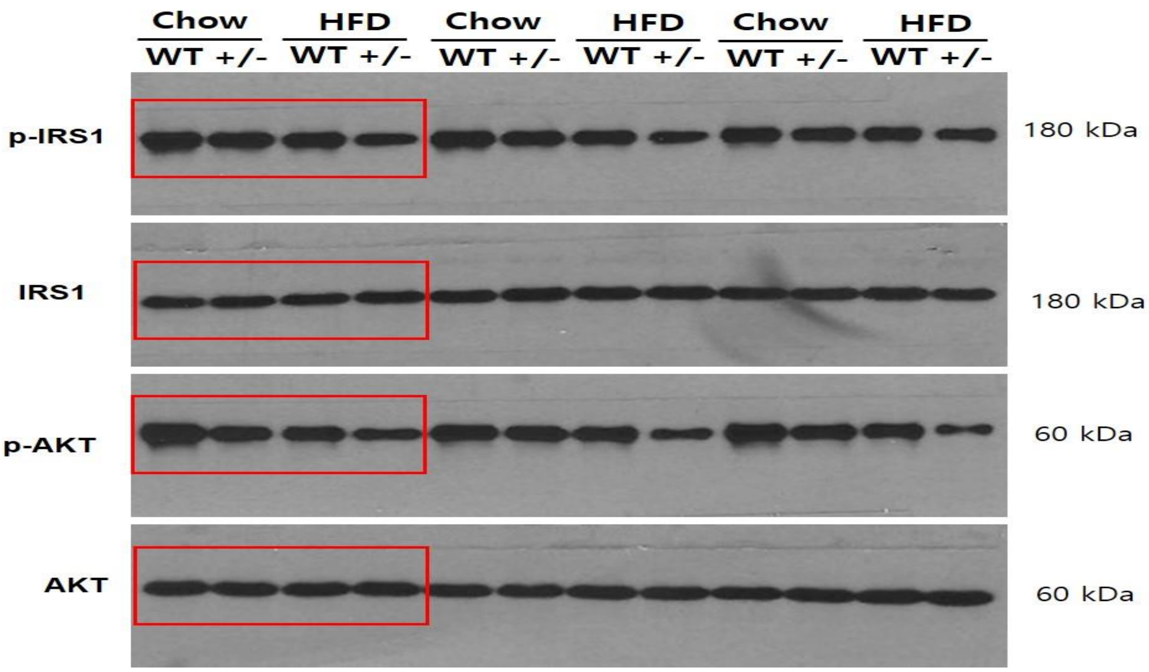

Supplementary Figure S4. Full length western blot membranes relative to Fig 4d. Red boxes indicate the cropping lines used to generate the figures.

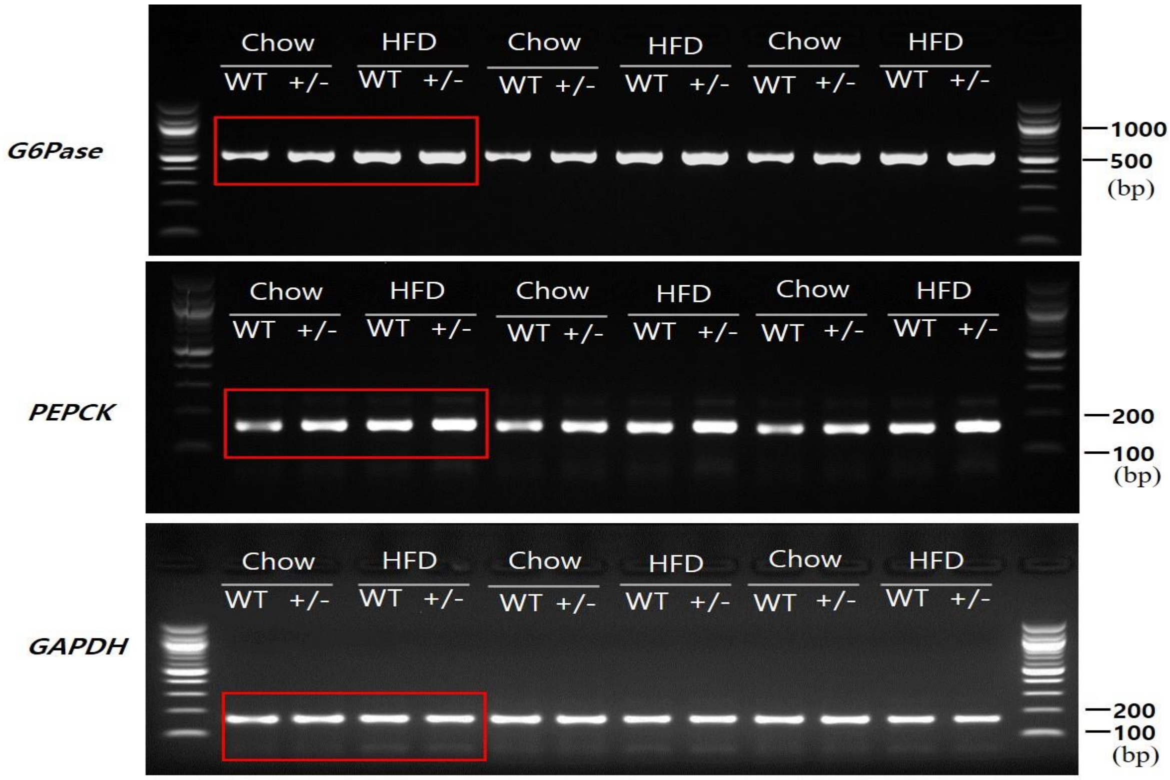

Supplementary Figure S5. Full length agarose gels relative to Fig 4e. Red boxes indicate the cropping lines used to generate the figures.

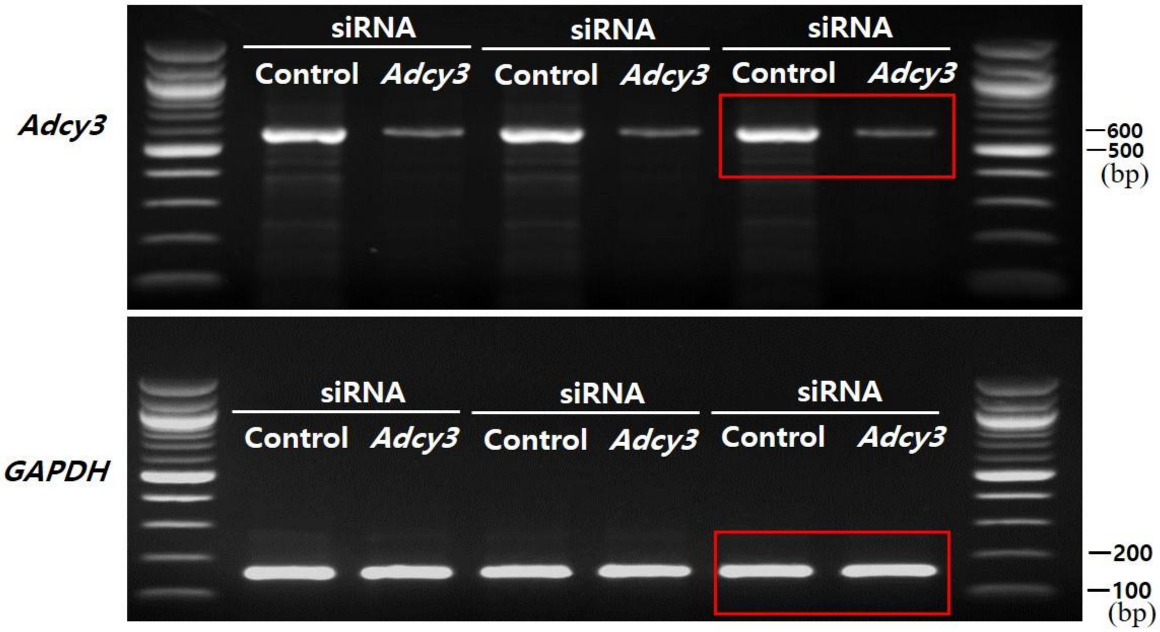

Supplementary Figure S6. Full length agarose gels relative to Fig 4f. Red boxes indicate the cropping lines used to generate the figures.

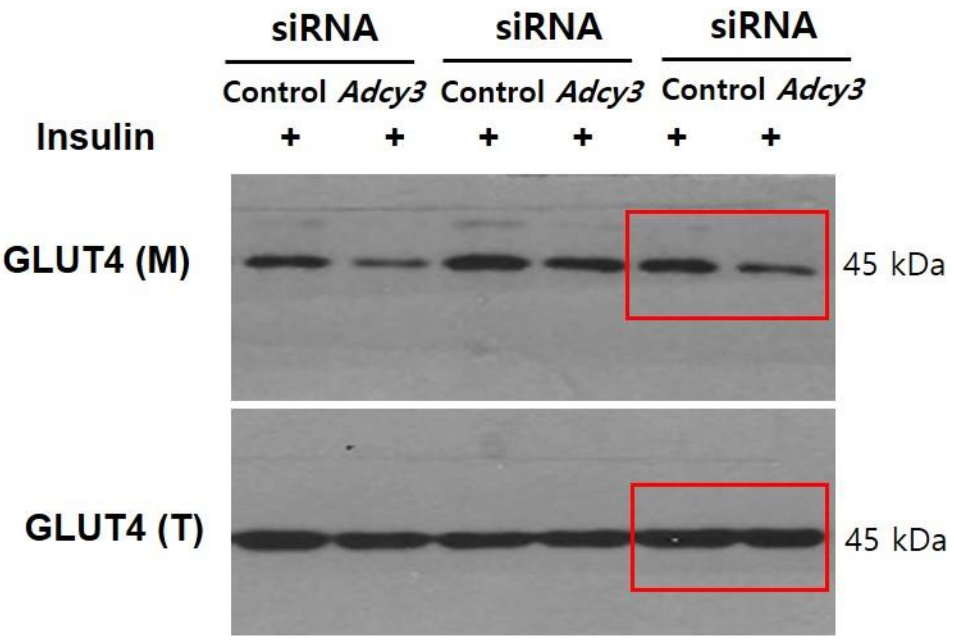

Supplementary Figure S7. Full length western blot membranes relative to Fig 4g. Red boxes indicate the cropping lines used to generate the figures.

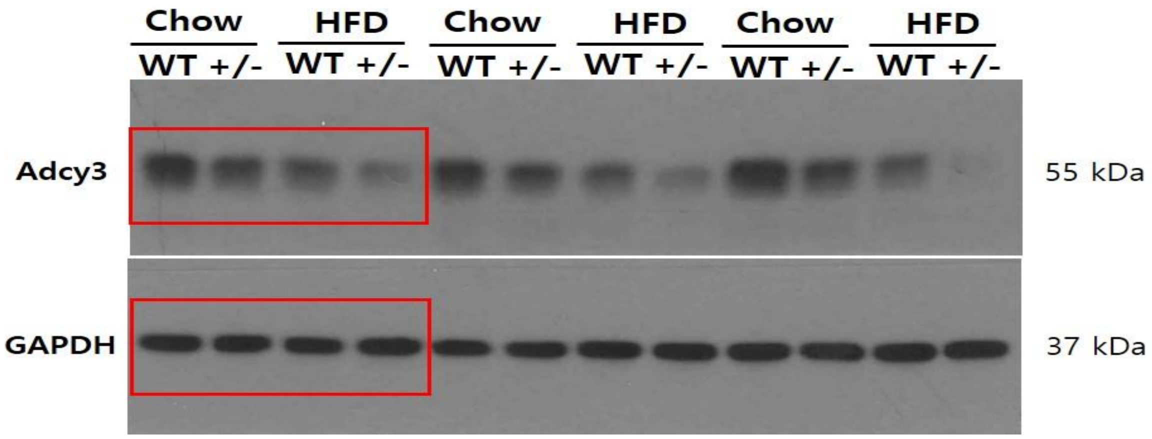

Supplementary Figure S8. Full length western blot membranes relative to Fig 5b. Red boxes indicate the cropping lines used to generate the figures.

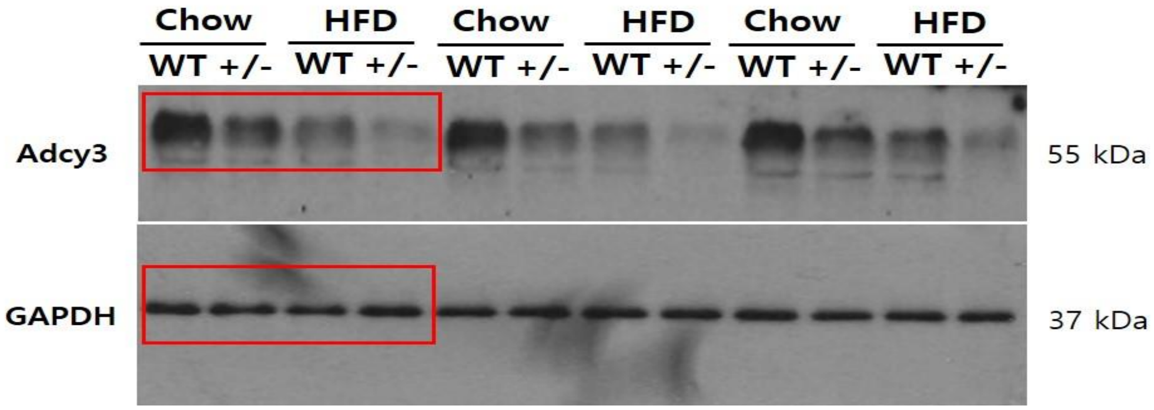

Supplementary Figure S9. Full length western blot membranes relative to Fig 5c. Red boxes indicate the cropping lines used to generate the figures.

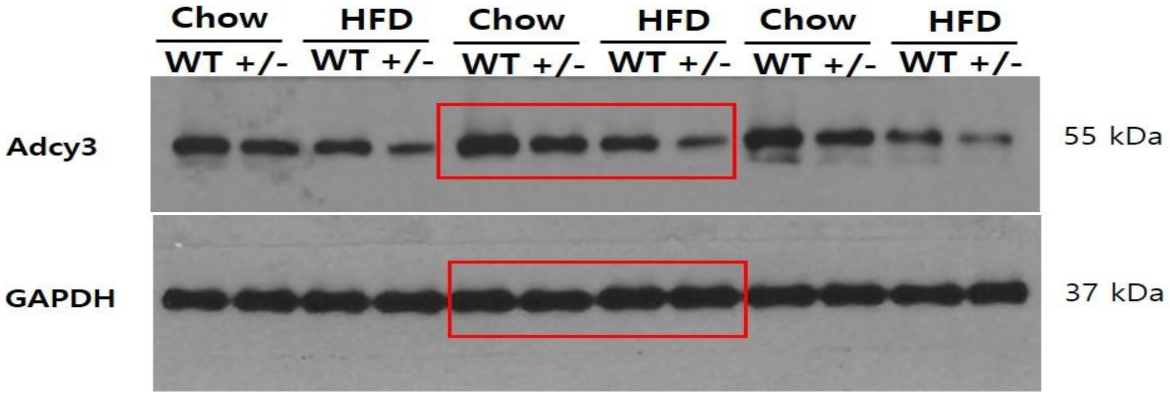

Supplementary Figure S10. Full length western blot membranes relative to Fig 5d. Red boxes indicate the cropping lines used to generate the figures.

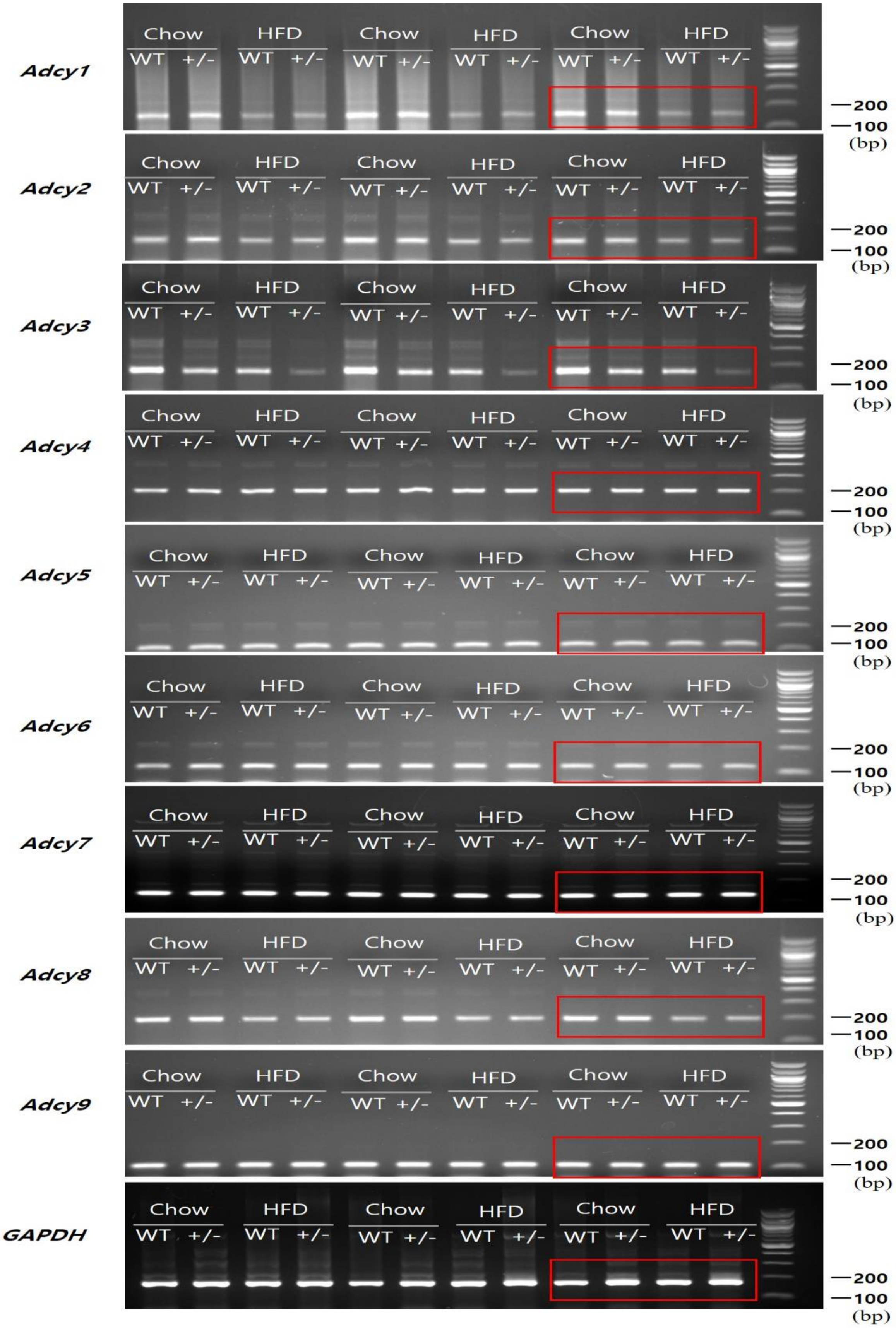

Supplementary Figure S11. Full length agarose gels relative to Fig 5e. Red boxes indicate the cropping lines used to generate the figures.

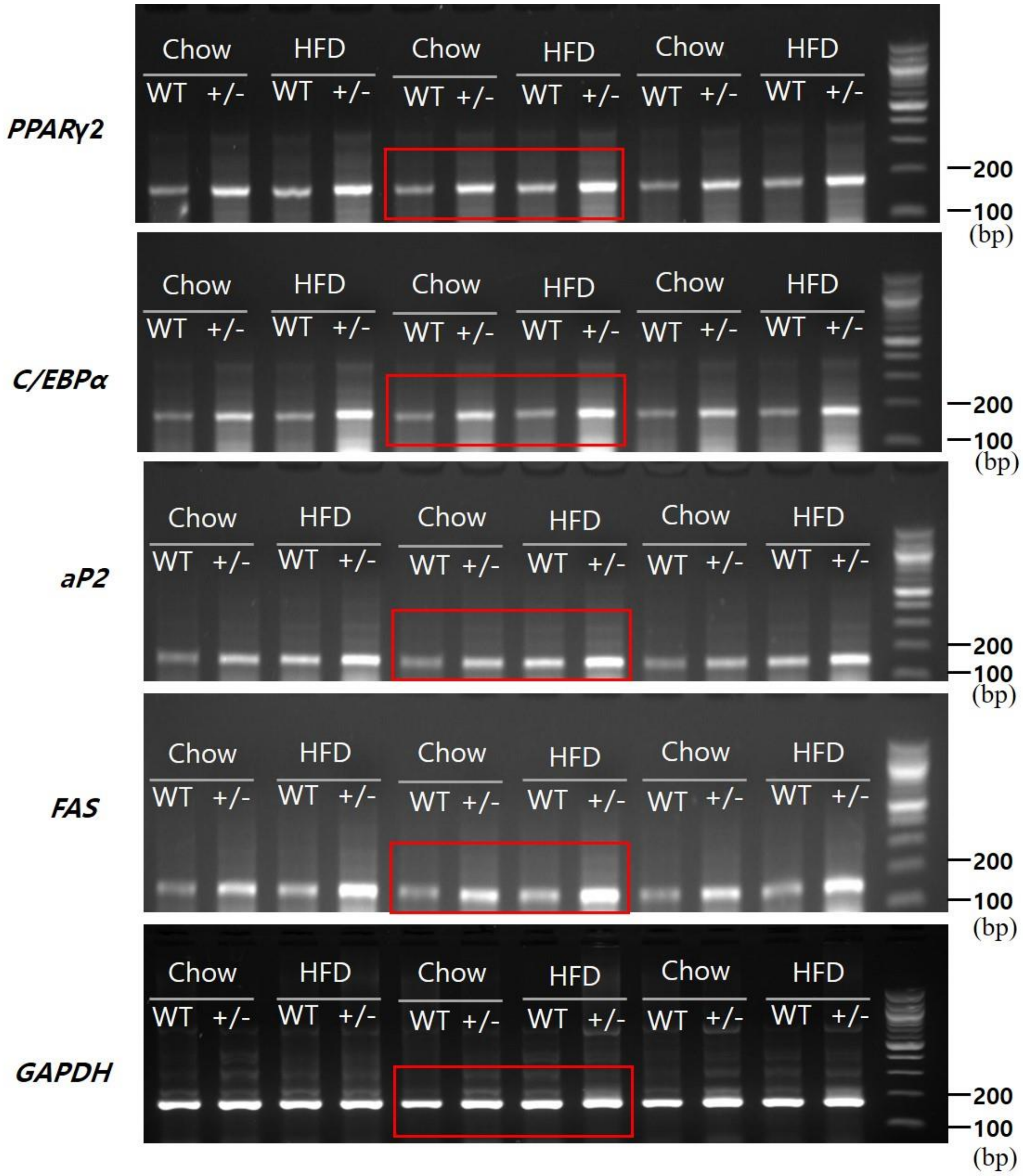

Supplementary Figure S12. Full length agarose gels relative to Fig 6a. Red boxes indicate the cropping lines used to generate the figures.

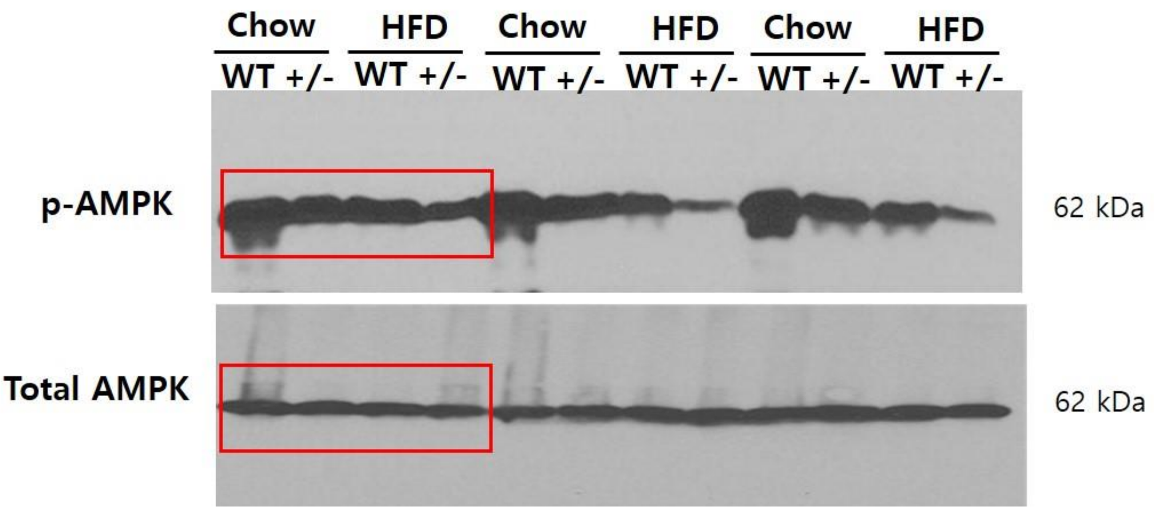

Supplementary Figure S13. Full length western blot membranes relative to Fig 6b. Red boxes indicate the cropping lines used to generate the figures.

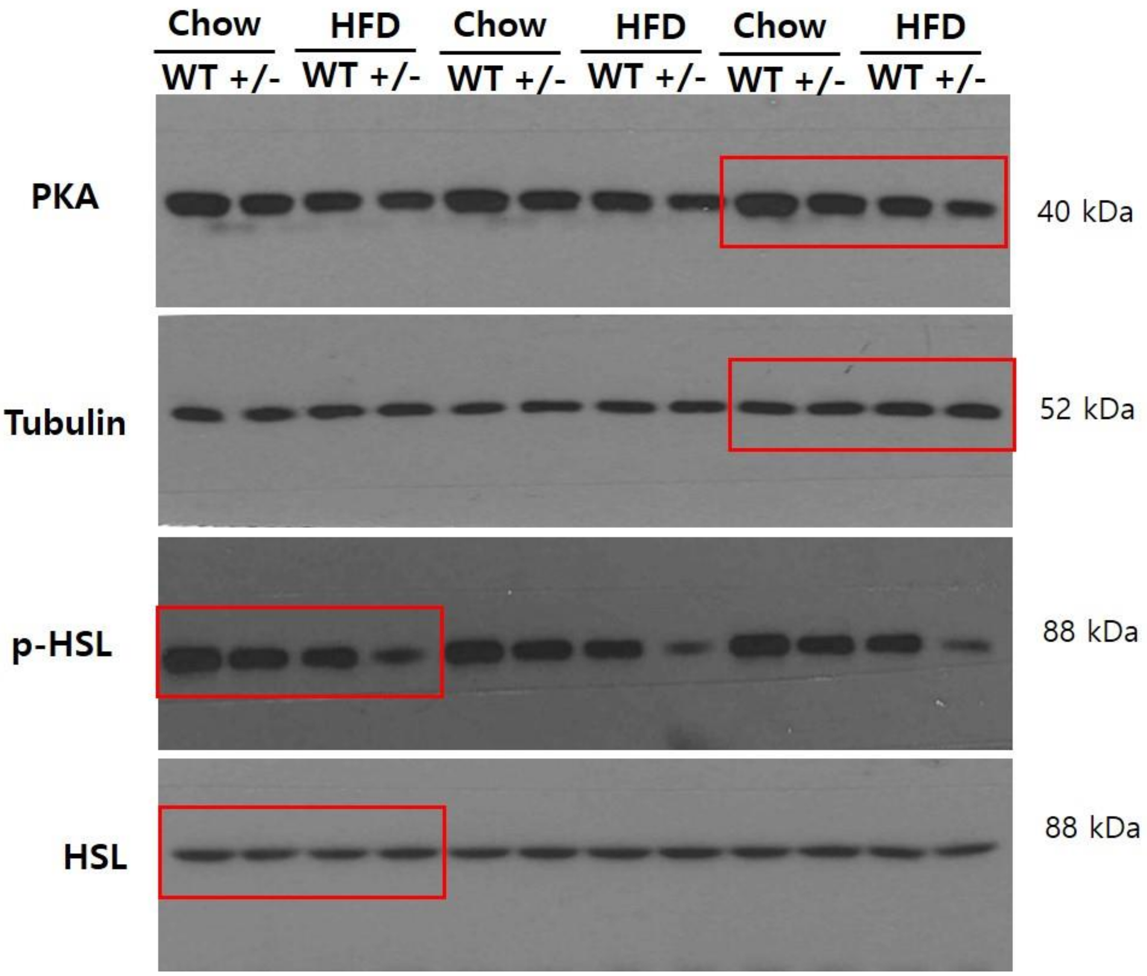

Supplementary Figure S14. Full length western blot membranes relative to Fig 6c. Red boxes indicate the cropping lines used to generate the figures.

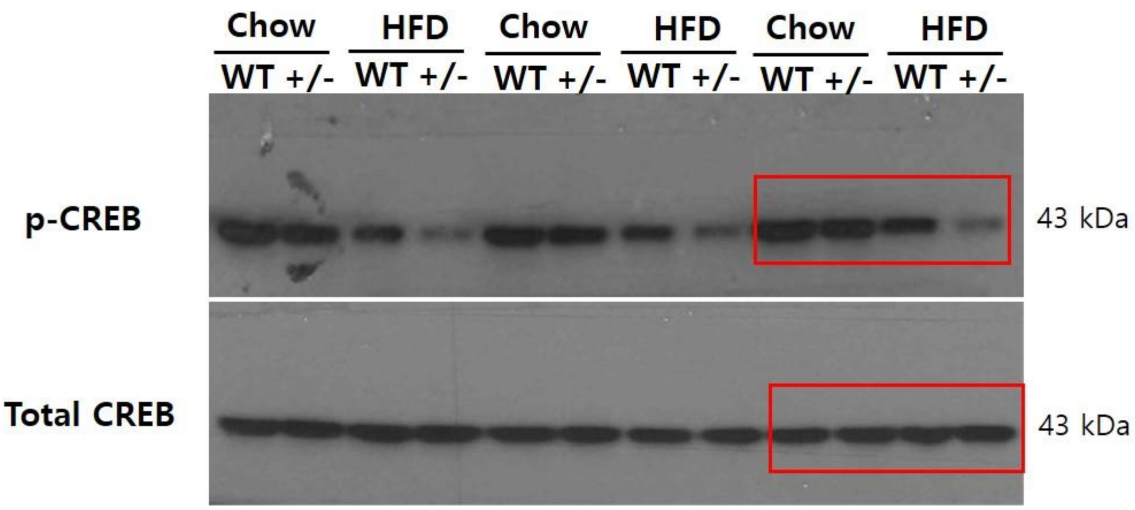

Supplementary Figure S15. Full length western blot membranes relative to Fig 6d. Red boxes indicate the cropping lines used to generate the figures.

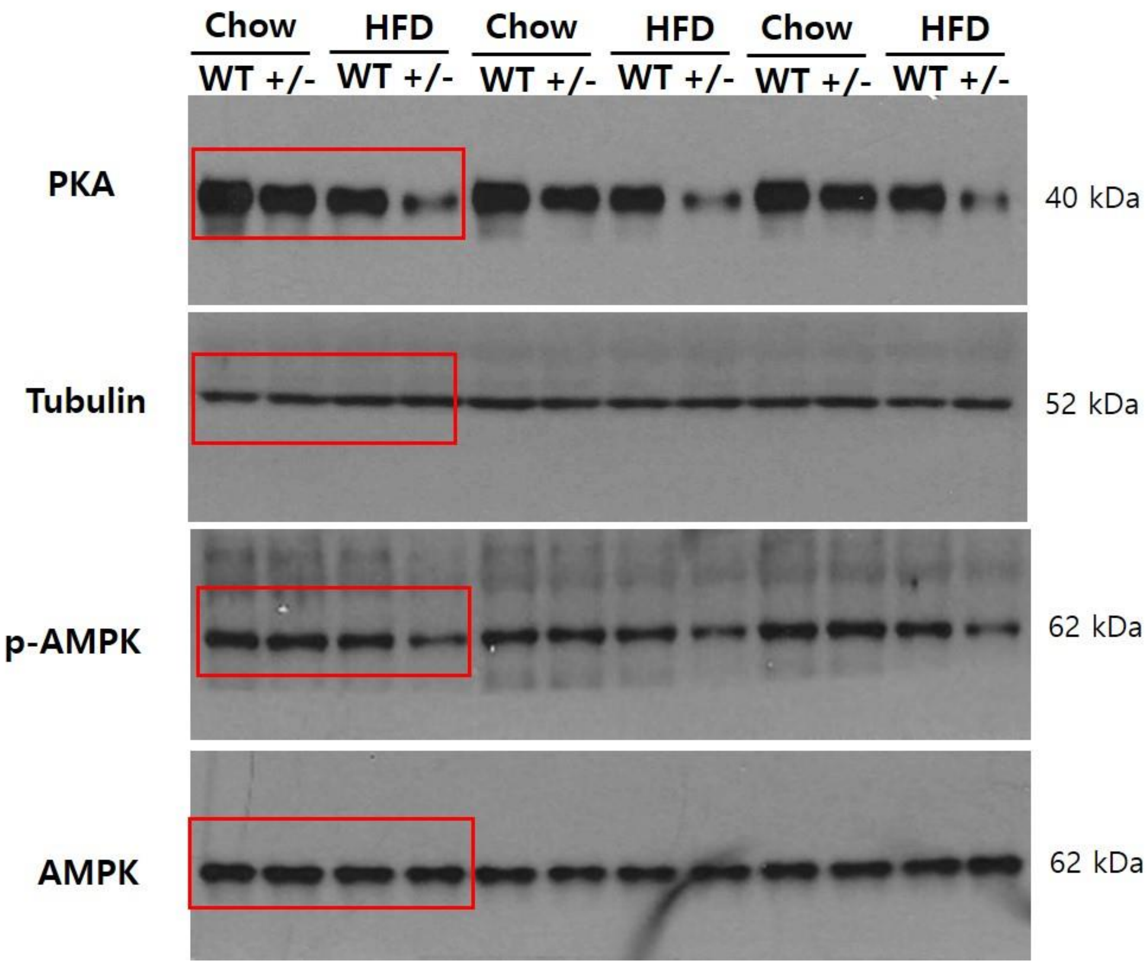

Supplementary Figure S16. Full length western blot membranes relative to Fig 6e. Red boxes indicate the cropping lines used to generate the figures.

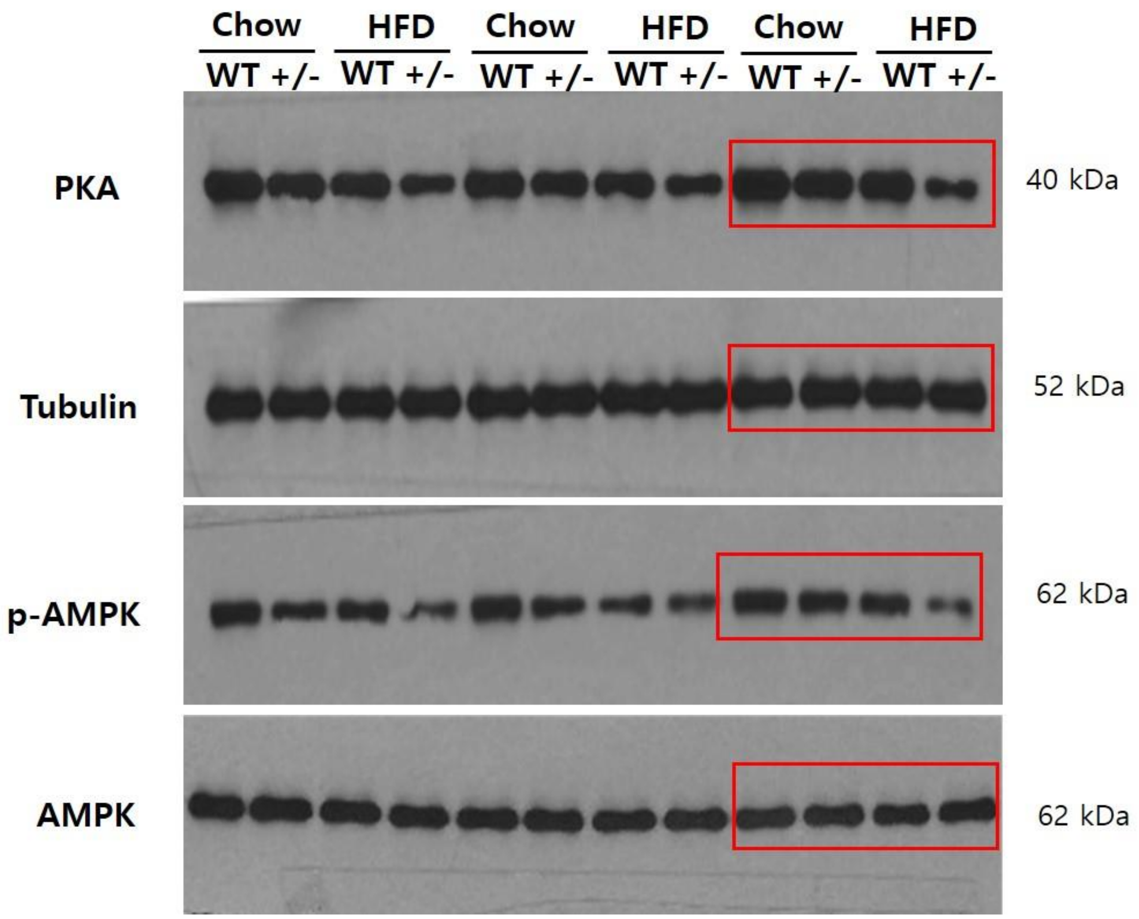

Supplementary Figure S17. Full length western blot membranes relative to Fig 6f. Red boxes indicate the cropping lines used to generate the figures.

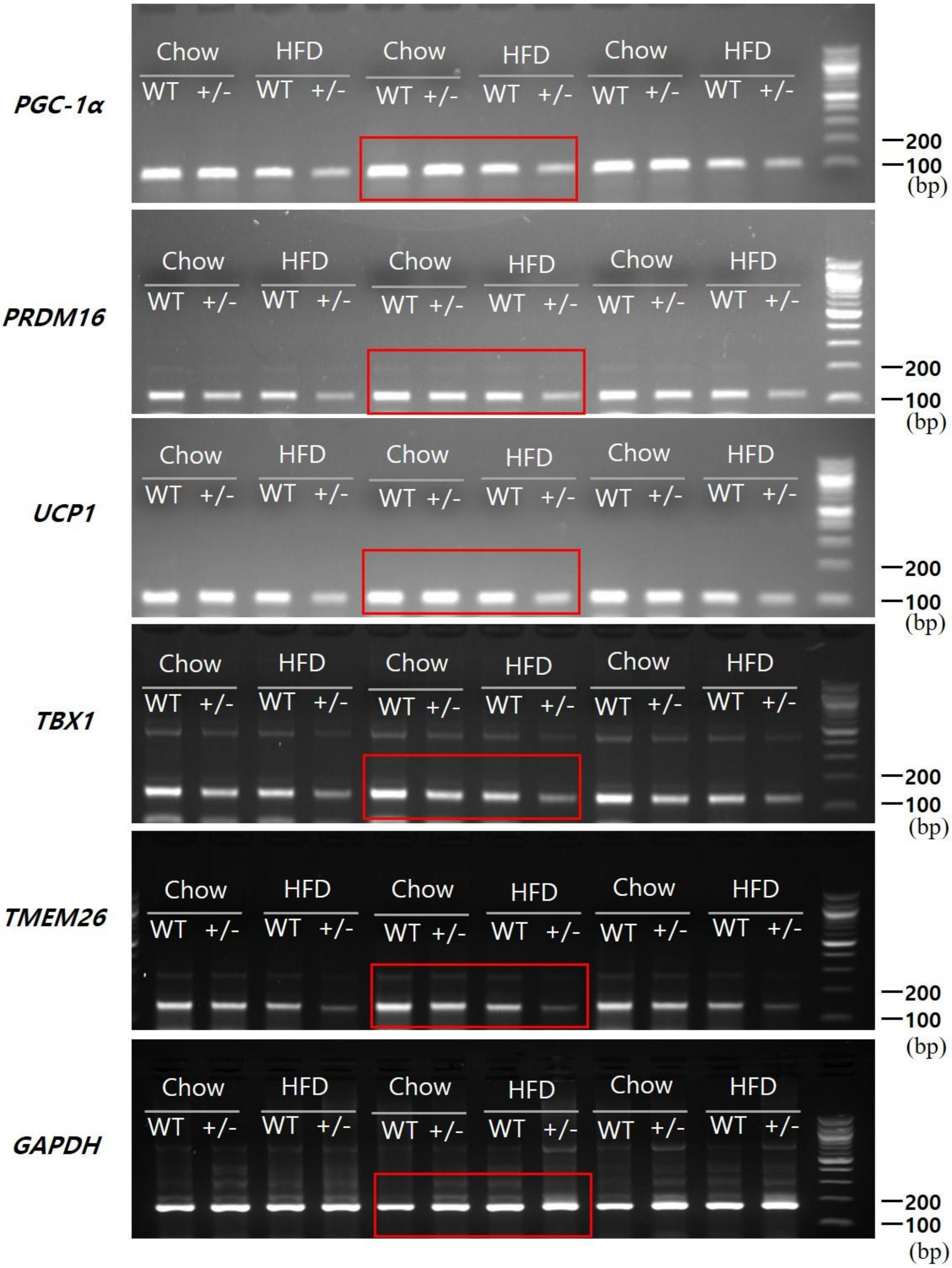

Supplementary Figure S18. Full length agarose gels relative to Fig 6g. Red boxes indicate the cropping lines used to generate the figures.

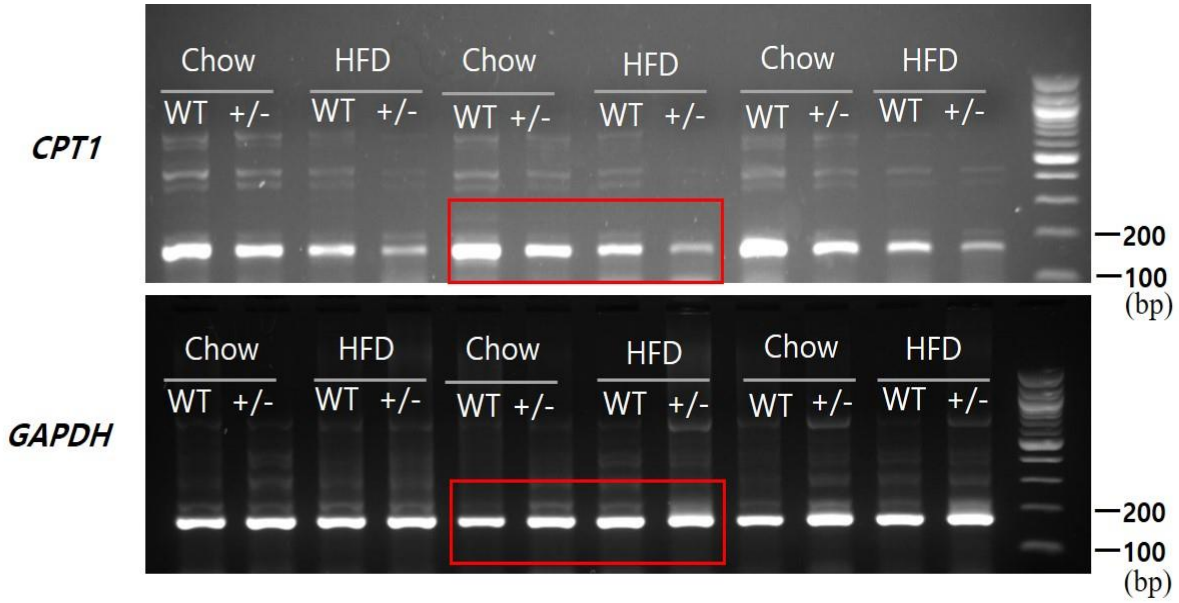

Supplementary Figure S19. Full length agarose gels relative to Fig 6h. Red boxes indicate the cropping lines used to generate the figures.

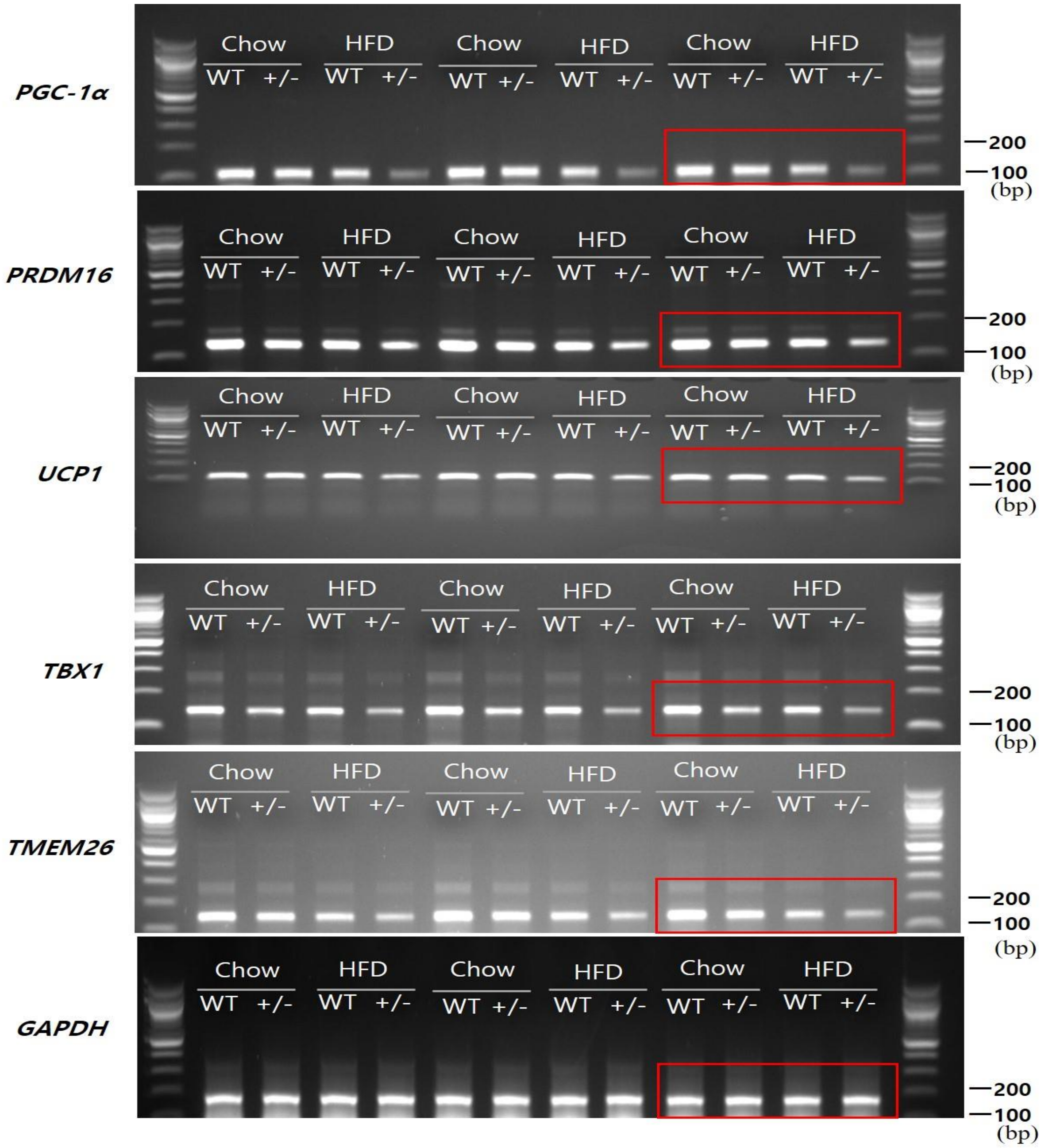

Supplementary Figure S20. Full length agarose gels relative to Fig 6i. Red boxes indicate the cropping lines used to generate the figures.
